# Comparative transcription profiles of *Candidatus* Accumulibacter and *Propionivibrio* under phosphate limitation in sequencing batch reactors

**DOI:** 10.3389/fmicb.2025.1650167

**Published:** 2025-10-28

**Authors:** Laëtitia Cardona, Pilar Natalia Rodilla Ramírez, Aline Adler, Christof Holliger

**Affiliations:** School for Architecture, Civil and Environmental Engineering, Environmental Engineering Institute, Laboratory for Environmental Biotechnology, Ecole Polytechnique Fédérale de Lausanne, Lausanne, Switzerland

**Keywords:** genome-resolved metatranscriptomics, phosphate-accumulating organism, glycogen-accumulating metabolism, ethylmalonyl-CoA pathway, methylmalonyl-CoA pathway, aerobic granular sludge

## Abstract

Polyphosphate-accumulating organisms (PAOs) play a crucial role in enhanced biological phosphorus removal (EBPR) processes. In addition to biosynthesis, they rely on phosphate for energy generation. However, *Candidatus* Accumulibacter, a model PAO, has been shown to adapt to low phosphate conditions by switching to a glycogen-accumulating metabolism (GAM), with variable success across genus members and experiments. This study aimed to explore the metabolic shift of several *Accumulibacter* species subjected to low-phosphate concentration in different operating conditions using metatranscriptomics analysis. Furthermore, the study enabled a comparison of the transcriptomic profiles of *Accumulibacter* with those of *Propionivibrio*, a glycogen-accumulating organism typically found in EBPR plants. Two sequencing batch reactors were operated with different carbon sources to enrich for different populations of *Accumulibacter*. After decreasing the influent phosphate concentration, carbon removal performance was maintained while anaerobic phosphate release dropped dramatically, suggesting a shift from a phosphate-accumulating to a glycogen-accumulating metabolism. Analysis of metatranscriptomics data indicated that *Accumulibacter regalis* (type I) and *Propionivibrio aalborgensis* remained the most abundant species after the phosphate decrease in the reactor with acetate-propionate and allylthiourea, while *Accumulibacter delftensis* (type I) and *Accumulibacter phosphatis* (type II) remained active in the reactor with acetate-glucose and no allylthiourea. Transcription of the genes from the ethylmalonyl-CoA pathway involved in the production of propionyl-CoA and regulation of the anaerobic redox balance was enhanced under low-phosphate conditions, especially for type I *Accumulibacter*. Conversely, the transcription of the methylmalonyl-CoA pathway was enhanced under low-phosphate conditions in *Propionivibrio* and type II *Accumulibacter*.

## 1 Introduction

Phosphorus plays a vital role in living organisms; however, when released into natural environments in high concentrations, it can be harmful through eutrophication of water bodies. Wastewater treatment plants partially remove phosphate through various methods, including excess sludge disposal, chemical precipitation, and biological treatment. The Enhanced Biological Phosphate Removal (EBPR) process, widely employed in biological wastewater treatment facilities, relies on phosphate-accumulating organisms (PAO) that store phosphate intracellularly. This specific microbial community is enriched through alternating phases of anaerobic feeding and aerobic starvation. During the anaerobic feeding phase, simple carbon compounds, such as acetate and propionate, are taken up by PAO and stored within cells as poly-*β*-hydroxyalkanoates (PHA). Polyphosphate (poly-P) and glycogen reserves provide the necessary energy and reducing equivalents, respectively, while inorganic phosphate (Pi) is released into the bulk liquid. In the subsequent aerobic-starvation phase, PHA is consumed for growth, and poly-P and glycogen reserves are replenished. Throughout this phase, the phosphate concentration in the liquid decreases as it is taken up by the cells (Seviour et al., 2003).

The removal effectiveness is largely influenced by the composition of the microbial community. Glycogen-accumulating organisms (GAO) have been considered to negatively impact the nutrient removal by competing with PAO for the carbon sources. Although GAO exhibits a metabolism similar to PAO, storing carbon under anaerobic conditions, they rely solely on their glycogen reserves for energy and reducing equivalents (Oehmen et al., 2007). Nevertheless, this population is frequently encountered in EBPR systems, and others have proposed that they, in fact, complement PAO in nutrient removal processes (Nielsen et al., 2019) and can cooperate with them for denitrification (Rubio-Rincón et al., 2017, 2019).

The best-described PAO in EBPR is *Candidatus* Accumulibacter (Petriglieri et al., 2022), referred to as *Accumulibacter* hereafter. As *Accumulibacter* is often used as a model to describe the PAO metabolism, it is often defined as a classical PAO, meaning the conversion of volatile fatty acids to PHA using polyphosphate and glycogen as energy and reducing equivalents sources. This contrasts with *Tetrasphaera*, another PAO often found in EBPR, especially in Denmark (Nielsen et al., 2019; Dueholm et al., 2022), which is thought to accumulate amino acids under anoxic conditions to provide energy to restore the polyphosphate reserve under oxic conditions (Nguyen et al., 2011; Singleton et al., 2022). Although highly investigated for PAO metabolism, a pure culture of *Accumulibacter* has not been successfully obtained. Until recently, the detection and classification of genus members were based on 16S rRNA gene and polyphosphate kinase *ppk1* sequences, respectively. *Accumulibacter* is a highly diversified group, and its species can be divided into different types (I and II), which have been further subdivided into clades. With the increase in available genomes, the taxonomic classification of *Accumulibacter* has become more precise through the phylogenetic analysis based on marker genes (Petriglieri et al., 2022). In addition to the taxonomy improvements, the development of omics technologies combined with batch tests has allowed for narrowing down the biochemical pathways involved in EBPR. The volatile fatty acids (VFA, acetate, and propionate) are anaerobically taken up *via* a proton acetate symporter (*actP*) activated by proton motive force (PMF). The PMF is generated by the combined efflux of proton-inorganic phosphate, from poly-P hydrolysis *via* the *pit* system (Saunders et al., 2007; Burow et al., 2008), which is commonly present in *Accumulibacter*, and in lesser proportion *via* the F1F0-ATPase (Qiu et al., 2020). The VFA is then transformed into PHA *via* multiple pathways (Tan et al., 2014). The ATP is provided by the conversion of the poly-P reserve to P_i_ either *via* the action of the polyphosphate kinase or the combined action of the AMP phosphotransferase and adenylate kinase (Welles et al., 2017). Although glycolysis is considered to be the primary source of reducing equivalents, other sources were suggested to balance models: the full tricarboxylic acid (TCA) cycle (Comeau et al., 1986), the combination of glycolysis with the left branch of the TCA cycle (Pereira et al., 1996), or the split TCA cycle combined with the anaplerotic route (pyruvate to oxaloacetate) (Hesselmann et al., 2000) or the use of the glyoxylate shunt (Yagci et al., 2003). The high heterogeneity in *Accumulibacter* species can partially explain the absence of consensus in defining a specific metabolism (Páez-Watson et al., 2024).

Previous studies have demonstrated the capacity of *Accumulibacter* to exhibit a GAO-like metabolism under high COD/P-PO_4_ ratios and introduced the concept of PAM and GAM for polyphosphate- and glycogen-accumulating metabolisms (Acevedo et al., 2012, 2017; Welles et al., 2015, 2017; Zheng et al., 2022). Welles et al. (2015) subjected three different *Accumulibacter*-enriched biomasses to phosphate-deprived conditions and demonstrated that (i) both type I and II were able to shift from PAM to GAM, (ii) type II was more efficient in shifting metabolism, probably due to a higher VFA uptake ability that could be derived from different glycolysis pathways, and (iii) type II exhibited a partial GAM when phosphate was low, but poly-P reserves were not limiting the VFA uptake (Welles et al., 2015).

The results of these different studies are based on stoichiometric analysis, including poly-P, glycogen, PHA, and VFA measures to determine the metabolism, and on Fluorescent *In Situ* Hybridization (FISH) analysis to estimate the proportion of the different *Accumulibacter* and GAO populations. However, pathways used by *Accumulibacter* while shifting metabolism are based on hypotheses derived from GAO, such as *Competibacter* or *Propionivibrio* (McIlroy et al., 2014; Albertsen et al., 2016), and potential differences between the GAM of *Accumulibacter* and the aforementioned GAOs or among *Accumulibacter* clades remain unknown. In order to deepen the understanding of the metabolism of *Accumulibacter*, genome-resolved metatranscriptomics analysis was carried out on *Accumulibacter*-enriched biomasses containing both type I and II under high- and low-phosphate conditions. Furthermore, the transcriptomic profile of *Accumulibacter* under low-phosphate conditions was compared with that of the co-occurring GAO *Propionivibrio*.

## 2 Materials and methods

### 2.1 Experiments operation

In order to study the metabolic transcription of different species of *Accumulibacter* under low-phosphate conditions, the results from two experiments were compared. The two reactors were operated apart from each other, with a different medium composition and an inoculum sampled from the same wastewater treatment plant, but at different moments.

In experiment RA, the carbon source came from acetate and propionate at equal proportions on a COD basis (COD 300 mg/L), and the nitrification was inhibited by adding allylthiourea in the medium. For 103 days, the COD/P-PO_4_ (mg/L COD/mg/L P-PO_4_) ratio was maintained at a value of 12. Then, the phosphate concentration in the medium was decreased to reach a COD/P-PO_4_ ratio of 200. After the phosphate reduction, a soft conditioning cycle was operated by inverting the settling and withdrawal phases with the aerobic phase for two cycles repeated twice in two weeks. The experiment was previously described in detail in (Cardona et al., 2025).

The experiment RC was operated for several years in the lab before being used for this experiment. Day 0 corresponds to the start of the present study. The carbon source came from acetate and glucose, and the nitrification was not inhibited in that experiment. After 76 days of operation, the COD/P-PO_4_ ratio was progressively modified from 20 to 200 for 27 days (day 76 to 103). Then, the initial COD/P-PO_4_ ratio of 20 was recovered and maintained for 28 days. A second decrease of the COD/P-PO_4_ ratio was initiated on day 131 and maintained for over 42 days. No conditioning cycle was operated in this experiment.

### 2.2 Reactor set-up

Both experiments were operated in a bubble column sequencing batch reactor (SBR) of 6.2 cm diameter and 2.4 L working volume in fill-draw mode. The temperature was regulated at 18 °C +/−1 °C by recirculating water in the double wall of the reactor. The pH was maintained at 7.5 +/− 0.5 by monitoring and regulating the injection of 1 mM HCl or 1 mM NaOH with an ISFET probe (Endress+Hauser, Switzerland) using a PID process control. Headspace gas recirculation was used to mix the biomass. Nitrogen or air was added to adjust for the concentration, and this was controlled *via* a PID to maintain an oxygen concentration of 0% under the anaerobic phase or 100% in the aerobic phase. The flow rate of the gas pump was set at 2 L/min. The pO_2_ was monitored by an ISFET probe (Endress+Hauser, Switzerland). Data collected by the reactor probes and the control of the different pumps were relayed to/from a computer through relay modules (WAGO, Switzerland) and processed using the software DAQFactory (AzeoTech, Inc.).

A typical cycle corresponded to 5 to 10 min of sparging nitrogen gas for RA and RC, respectively, 60 min of feeding under anaerobic mixing conditions from the bottom of the reactor; 30 min of anaerobic phase; 120 min of aerobic phase by sparging compressed air; 15 min of nitrogen sparging for RA exclusively; 10 min of settling and withdrawal of half of the reactor working volume. The hydraulic retention time was set at 9.5 h, and the solid retention time was set at 21 days by sampling the mixed liquor three times a week at the end of the aerobic phase. The sampling was also adjusted according to the biomass concentration in the reactor to avoid a washout of the biomass. The experiment setup and design are described in the Supplementary Figure 1.

### 2.3 Inoculum and media composition

The sludge used as inoculum was collected in the anaerobic tank of the Thunersee wastewater treatment plant (Thun, Switzerland), which performs biological phosphorus removal.

For experiment RA, the reactor influent was created by mixing two 8.89 times concentrated solutions of C and NP and Milli-Q water. Concentrated solution C contained 5.67 g/L C_2_H_3_O_2_Na-3H_2_O, 2.28 g/L C_3_H_5_O_2_Na, 0.889 g/L MgSO_4_-7H_2_O, 2.2 g/L MgCl_2_-6H_2_O, and 0.4 g/L CaCl_2_-H2O. Concentrated solution NP contained 1.671 g/L K_2_HPO_4_, 0.649 g/L KH_2_PO_4_, and 0.048 g/L C_4_H_8_N_2_S to inhibit the nitrification and 50 mL of trace elements solution composed of 16.22 g/L C_10_H_14_N_2_Na_2_O_8_-H_2_O, 0.44 g/L ZnSO_4_-7H_2_O, 1.012 g/L MnCl_2_-4H_2_O, 7.049 g/L (NH_4_)_2_Fe(SO_4_)2-6H_2_O, 0.328 g/L (NH_4_)_6_Mo_7_O_24_-4H_2_O, 0.315 g/L CuSO_4_-5H_2_O, and 0.322 g/L CoCl_2_-6H_2_O. Both solutions were autoclaved in 10 L glass bottles. Before use, 250 mL of bicarbonate solution composed of 0.933 g/L NH_4_HCO_3_ and 0.533 g/L KHCO_3_ was added to the NP solution to reach a final volume of 10 L. At each cycle, 120 mL of concentrated solutions C and NP were mixed with 960 mL of distilled water to feed the reactor and achieve a final chemical oxygen demand (COD) concentration of 300 mgO_2_/L in the SBR.

For experiment RC, the synthetic wastewater contained a COD: NH_4_-N: PO_4_-P ratio of 400:28:20 mg/L. The final medium resulted from mixing two 24-times concentrated solutions with milli-Q water. Concentrated solution A contained the carbon sources with 11.6 g/L acetate (C_2_H_3_O_2_Na-3H_2_O), 4.9 g/L glucose (C_6_H_12_O_6_-H_2_O), 2.1 g/L MgSO_4_-7H_2_O, 0.3 g/L CaCl_2_-2H_2_O, 0.8 g/L KCl, 2.5 g/L NH_4_Cl, 0.02 g/L yeast extract, and 7 mL trace elements solution. The solution was autoclaved without the glucose that was prepared aside, filtered, and added to the main solution under a laminar flow hood. Concentrated solution B contained the phosphorous source of 2.7 g/L, 0.6 g/L, or 0.2 g/L K_2_HPO_4_, for a COD/ P-PO_4_ ratio of 20, 90, and 200, respectively.

### 2.4 Nutrient performance monitoring

The nutrient removal performance of the reactors was measured on a weekly basis. Samples of 50 mL were taken from the middle of the water column at the end of the anaerobic and aerobic phases, and centrifuged for 5 min at room temperature at 4200 x g. The supernatant was filtered (0.45 μm). A sample of the synthetic reactor influent was also collected and filtered (0.45 μm). The samples were stored at 4 °C until further analysis was conducted. The concentration of the anions (P-PO_4_^3−^, N-NO^3−^, and N-NO^2−^) was measured using ionic chromatography (IC, ICS-90, IonPacAS14A column) with an electrical conductivity detector (Dionex, Switzerland). The chemical oxygen demand was measured by spectrophotometry using two different kits: LCK514 (100–2000 mgO_2_/L) and LCK314 (15–150 mgO_2_/L) (Hach, USA), measured on a spectrophotometer DR 3900 (Hach, USA).

The total and volatile solids were determined in the sludge obtained by centrifuging 100 mL of the mixed liquor reactor sample taken at the end of the aerobic phase. The mass of the dried pellet after 12 h of drying at 105 °C yielded the total solids, and the mass loss after 2 h of calcination at 550 °C resulted in volatile solids. The results of the monitoring are summarized in the Supplementary Table 1.

### 2.5 Metatranscriptomics sampling and extraction

For the metatranscriptomics analysis, biomass samples were collected just before the phosphate concentration was changed and again when the anaerobic release of phosphate ceased. One sample was taken after 15 min of the anaerobic feeding started, and another 15 min after the aerobic phase started. Samples were collected during three consecutive cycles for each time point, leading to a total of 12 samples per experiment. An aliquot of 15 mL of mixed liquor was sampled, put on ice, and quickly centrifuged for 1 min at 4 °C and 4,200 x g. The pellet was resuspended in 2 volumes (g pellet:ml volume) of RNA protect Tissue (Qiagen, Germany) to protect the RNA from degradation and homogenized by passing 3 times through a needle (26G). After an incubation at room temperature (RT) for 5 min, the sample was centrifuged for 5 min at RT and 5,000 x g, and the supernatant was discarded. The pellet was snap-frozen in liquid nitrogen and stored at −80 °C until RNA extraction was performed. The RNA preservation was tested beforehand to evaluate the degradation and quantity of RNA after a long period of storage, and showed good preservation capacity (data not shown).

The protocol for the RNA extraction was described in Cardona et al. (2025). Briefly, the samples were resuspended in 0.5 mL of TRIzol (#15596–0026, Invitrogen, Fisher Scientific AG, Switzerland) and incubated for 5 min at RT. Then, 0.1 mL of chloroform 99 + % was added, and the mixture was vortexed for 15 s and incubated for 2 min at RT before being centrifuged for 15 min at 15500 x g at 4 °C. The upper portion was recovered and mixed with 400 μL of 100% ethanol. RNA was purified using an RNA purification kit (Direct-zol RNA Miniprep #R2050, Zymo Research, Germany) following the manufacturer’s recommendations, except that centrifugation was performed for 1 min at 13000 x g. DNA was removed using a TURBO DNA-free™ kit (#AM1907, Thermo Fisher Scientific, Switzerland) following the manufacturer’s recommendations. RNA was purified by adding 76 μL magnetic beads (Agencourt RNA Cleaner XP, #A63987, Beckman Coulter) to the extracted RNA. The RNA was washed by alternating three times between the addition of 70% ethanol solution and removal after 10 min on a magnetic rack. After removing the ethanol, 32 μL of RNAse and DNase-free water was added to the pellet out of the rack and resuspended 10 times by up and down. The samples were incubated for 1 min before being returned to the rack for 1 min. Finally, the supernatants were collected. The quality of the extraction and the absence of DNA on the RNA-extracted samples were assessed by carrying out a PCR. The reaction mix was composed of 2 μL of 5X MyFi reaction buffer and 0.4 μL of the following primers: 27F-FTCGTCGGCAGCGTCAGATGTGTATAAGAGACAGAGMGTTYGATYMTGGCCTCAG and 338R-GTCTCGTGGGCTCGGAGATGTGTATAAGAGACAGGCTGCCTCCCGTAGGAGT (Microsynth, Switzerland), 0.4 μL of MyFi DNA polymerase (#BIO25049, Labgene Scientific SA, Switzerland), and 5.8 μL of nuclease-free demineralized water. The PCR program was composed of the following steps: 95 °C for 1 min, 30 cycles of 15 s at 95 °C, 15 s at 56 °C, and 15 s at 75 °C. The PCR products were checked on an agarose gel of 1.5% with a migration at 100 V. Bacterial ribosomal RNA (rRNA) was removed using a QiaSeqFastSelect 5S/16S/23S kit (#335925, Qiagen) following the manufacturer’s recommendations on 1 μg of total RNA (protocol with TruSeq® stranded library preparation) with the following modification: The first step of combined fragmentation and hybridization was performed for 1 min at 89 °C. Libraries were then generated using the TruSeq Stranded mRNA sample preparation kit (#20020594, Illumina, USA) and IDT for Illumina TruSeq RNA UD Indexes (#20022371, Illumina) following the reference guide #1000000040498 for the LS procedure without optional steps. For the clean-up amplified DNA step, the ratio of magnetic beads to PCR products was 0.7, and 20 μL of RSB was added to release the genetic material from the beads. The amplification was quantified with the Qubit dsDNA HS Assay Kit (#Q32854, Life Technologies), and the quality was checked by electrophoresis using the Agilent High Sensitivity DNA Kit (# 5067–4,626, Agilent Technologies). The concentrations of the samples were normalized to 10 nM and pooled. Sequencing analysis was performed at the Lausanne Genomic Technologies Facility, University of Lausanne (Switzerland), on a NovaSeq 6,000 in paired-end mode (2 × 150). A sequencing run was carried out for each experiment separately.

### 2.6 Metagenome database construction

Metagenome-assembled genomes (MAGs) of *Accumulibacter* and *Propionivibrio* were obtained from public databases (Martín et al., 2006; Flowers et al., 2013; Skennerton et al., 2015; Welles et al., 2015; Kantor et al., 2015; Albertsen et al., 2016; Parks et al., 2017; Zhang et al., 2017; Arumugam et al., 2019, 2021; Camejo et al., 2019; Ye et al., 2020; Singleton et al., 2021; Lin et al., 2021; McDaniel et al., 2021b, 2021a; Xie et al., 2024a, 2024b) and previous research done by our team (Adler et al., 2022; Saini et al., 2024). The nomenclature and classification in the present study are based on this latest reevaluation (Petriglieri et al., 2022). The quality of the genomes was assessed using CheckM [Parks et al. (2015), v1.2.2] and their average nucleotide identity between them using FastANI [Jain et al. (2018), v1.33]. The genomes were dereplicated based on the results of fastANI, considering 97% as a threshold to separate two genomes. Supplementary Table 2 summarizes the information related to the MAGs, and Supplementary Table 3 contains the fastANI matrix result. A representative genome for each group was selected based on its quality (completeness, contamination, and fragmentation) and the presence of universal marker genes, obtained using fetchMGs (https://github.com/motu-tool/FetchMGs, v1.3) (Supplementary Table 4). DRAM v1.4.6 (Shaffer et al., 2020) and eggNOG-mapper v2.1.11 (Cantalapiedra et al., 2021) were used for gene prediction and annotation. The selected genomes were used as a database for the metatranscriptomics mapping. The annotation file is presented in the Supplementary Table 5.

### 2.7 Genome-resolved metatranscriptomics analysis

The quality of the reads was evaluated using FastQC v0.11.9 (https://www.bioinformatics.babraham.ac.uk/projects/fastqc/) at each step of the analysis pipeline. The results were summarized using MultiQC v1.13 (Ewels et al., 2016). Reads were filtered and trimmed using BBDuk from BBMap v39.01 (https://jgi.doe.gov/data-and-tools/software-tools/bbtools/bb-tools-user-guide/bbmap-guide/) using the following parameters: ktrim = r, k = 23, mink = 11, hdist = 1, tpe, tbo for the adapter trimming steps and qtrim = rl, trimq = 20, minlen = 50, maq = 20, maxns = 1 for the quality trimming and filtering. Ribosomal RNA was removed using sortMeRNA [Kopylova et al. (2012), v4.3.6] using the databases for silva-bac-16 s-id90, silva-arc-16 s-id95, silva-euk-18 s-id95, silva-bac-23 s-id98, silva-arc-23 s-id98, silva-euk-28 s-id98, rfam-5 s-database-id98, and fam-5.8 s-database-id98 from Silva (Quast et al., 2012) and rfam (Kalvari et al., 2021). The remaining messenger RNA was mapped onto the metagenomes database of *Accumulibacter* and *Propionivibrio* described in section 2.6, using bowtie2 [Langmead and Salzberg (2012), v2.4.1] and the following arguments: very-sensitive mode, X 1000, phred33, and k 35, allowing to search for a maximum of 35 valid alignments for each read, considering a possible multi-mapping in the different MAGs of the database. The alignments were filtered out if the percentage of identity was lower than -p 95 using msamtools (https://github.com/arumugamlab/msamtools, v1.1.0). Supplementary Figure 2 represents the analytical pipeline, and Supplementary Figure 3 and Supplementary Table 6 summarize the number of reads kept at each step. FeatureCount [Liao et al. (2014), v2.0.1] was used to summarize the mapping results into a count table using the following parameters: t CDS, −g ID, -O, −M, -B, −-primary and -Q 2 to account for the uniquely mapping reads only (Supplementary Tables 7, 8 for RA and RC, respectively), discarding the multi-mapping reads from the subsequent analyses.

To ensure that no PAOs or GAOs genomes were missing from the mapping on the selective database, the unmapped reads were blasted using DIAMOND [v2.1.9.163, Buchfink et al. (2021)] in blastx mode (−k 1) to the Swiss-Prot and TrEMBL databases [2024_04, Bairoch (2000)] where the protein sequences with an annotation score of 2, 3, 4, and 5 were selected. The results for experiment RC showed that between 54 to 74% of the reads mapped to the Bacteria domain and 24 to 42% to the Eukaryota domain, whereas really few reads were assigned to viruses and Archaea. From the Bacteria domain, few reads mapped to some GAOs, *Contendobacter* (around 1%), *Competibacteraceae* (1.14%), and *Propionivibrio* (3%). For experiment RA, the unmapped reads were assigned 31% to the Eukaryota domain and 65% to the Bacteria domain. From the Bacteria domain, around 5.6% of the reads mapped to *Accumulibacter* sp. and 5.6% to *Contendobacter* but no other PAO or GAOs were identified with a relative abundance higher than 1%. Although some reads mapped to some PAOs and GAOs that were not included in our database, the percentage of mapping was really low, and it was decided to pursue the analysis without adding these genomes.

All the subsequent analyses were conducted using R (v4.3.3) on RStudio (2023.12.1 + 402). A Venn diagram was done using InteractiVenn (Heberle et al., 2015).

The transcription profile of each MAG in the different samples was evaluated by comparing the number of transcribed genes to the sum of counts per MAG and sample. MAGs with a high transcription level in each sample were selected to compare their transcription profile between different conditions or between different MAGs.

On selected MAGs, the low counts were filtered out using filtrExpr from EdgeR with the parameter min.count = 15 [Robinson et al. (2010), v4.0.16]. Then, the zero values were imputed by 0.5, and the counts were normalized by the gene length. Finally, a normalization using universal marker genes was done as described in Salazar et al. (2019), with the difference that the normalization was done at the MAG level. Briefly, each gene count of each MAG was divided by the median count of 10 universal marker genes of the same MAG, previously identified by fetchMGs. The advantage of using the universal marker genes, genes constitutively transcribed in any conditions, compared to more traditional methods, is the estimation of the transcription to the relative number of copies per cell of each MAG, as described by (Sunagawa et al., 2013; Milanese et al., 2019; Salazar et al., 2019). The data obtained was transformed into counts by dividing each value by the maximal value of each MAG and multiplying by 10^^9^.

Differential gene expression analysis compared the transcription level of one MAG between two conditions (high versus low influent phosphate concentration). In that case, DESeq2 [Love et al. (2014), v1.42.1], edgeR and limma [Ritchie et al. (2015), v3.58.1] were used, and a gene was defined as differentially expressed if it was determined as such in at least two of these methods with an adjusted *p*-value (Benjamini-Hochberg method) lower than 0.01 and no restriction on the log fold change value.

The transcription profile was also compared between two MAGs, *Propionivibrio aalborgensis* versus *Accumulibacter regalis* in the case of experiment RA, and *Accumulibacter delftensis* (type I) and *Accumulibacter phosphatis* (type II) in experiment RC. For these comparisons, the counts were summed at the KEGG Orthologs (KO) level per MAG and sample, and edgeR was used to identify the differential gene transcription (adjusted *p*-value lower than 0.01). For all analyses, comparisons were made for each phase separately (feeding and aerobic). A list of genes of interest is provided in the Supplementary Table 10. The results of the differential gene transcription analyses are provided in Supplementary Tables 11, 12, for RA and RC, respectively.

## 3 Results

### 3.1 Nutrient removal performances under high- and low-phosphate conditions

In two experiments (RA and RC), aerobic granular sludge reactors with biomass enriched in PAOs were switched to an influent low in phosphate. In experiment RA, the carbon sources were acetate and propionate, whereas in experiment RC, acetate and glucose were supplied.

**Figure 1:**
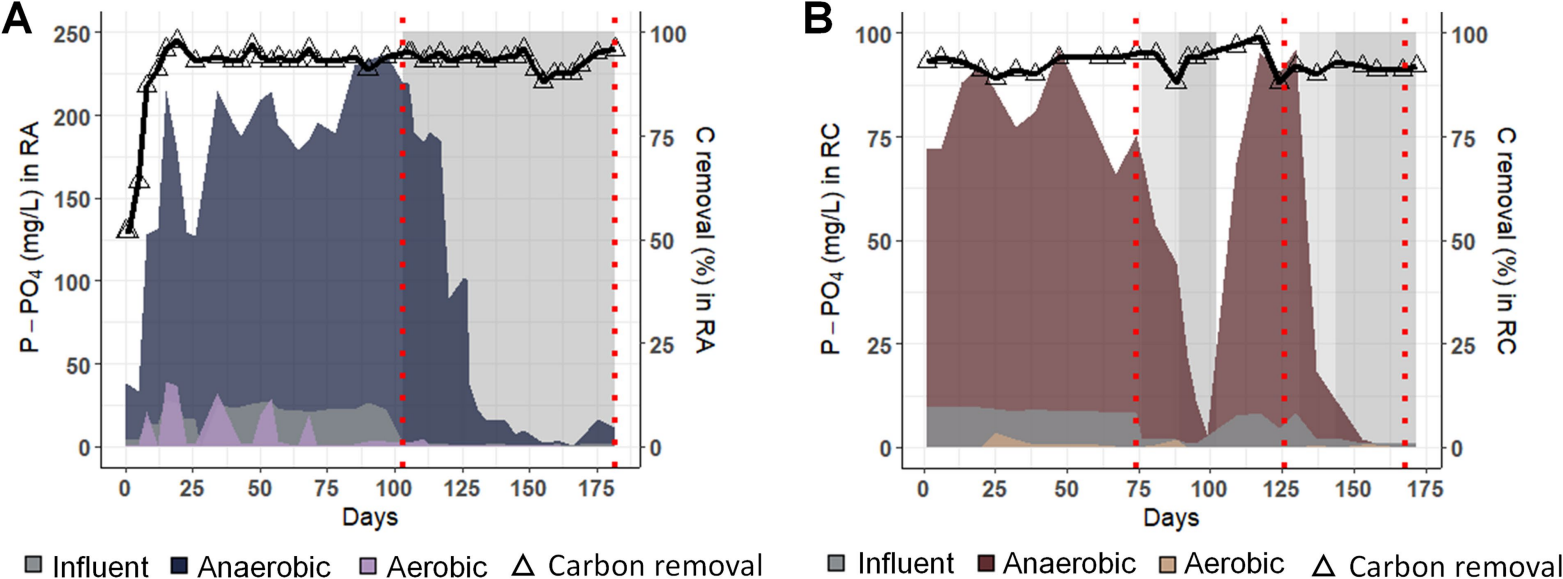
Nutrient removal performances for experiments RA and RC. Phosphate concentration in the influent (P-IN), at the end of the anaerobic (P-AN) and end of the aerobic phase (P-AE) and carbon removal efficiency (%) at the end of the feeding phase (right axis, represented by a triangle and a black line) **(A)** for the RA experiment and **(B)** for the experiment RC. Gray zones indicate the period with decreased phosphate concentration in the influent. Dashed red lines indicate the metatranscriptomics sampling points.

Before changing the influent phosphate (P-IN), the phosphate concentration at the end of the anaerobic phase (P-AN) reached a maximum of 237 and 96.5 mg/L. It decreased during the aerobic phase (P-AE) to as low as 0.80 mg/L and below the limit of detection for RA and RC, respectively (Figure 1).

Once P-IN was reduced to reach a COD/ P-PO_4_ ratio of 200, P-AN decreased in both experiments. In RA, from day 150, P-AN decreased to 1 mg/L until day 169, from which P-AN slightly increased to a maximum of 16.6 mg/L. In RC, while P-AN dropped to 1 mg/L in two weeks after the first modification (days 76 to 100), the decrease took less than a week in the second modification (days 130 to the end). The return to the initial P-IN, from days 100 to 130, led to an increase of P-AN of around 100 mg/L, similar to the concentration at the beginning of the experiment.

In both experiments, the carbon removal performance at the end of the anaerobic phase remained higher than 80% irrespective of the P-IN concentration. This result suggests a GAM phenotype, as phosphate release was low, while the carbon was taken up under anaerobic conditions. In order to evaluate the influence of the phosphate concentration on the PAO populations and their metabolism, metatranscriptomics samples were taken before and after P-IN reduction.

### 3.2 Metagenome-assembled genome (MAG) sequencing depth in metatranscriptomics

**Figure 2:**
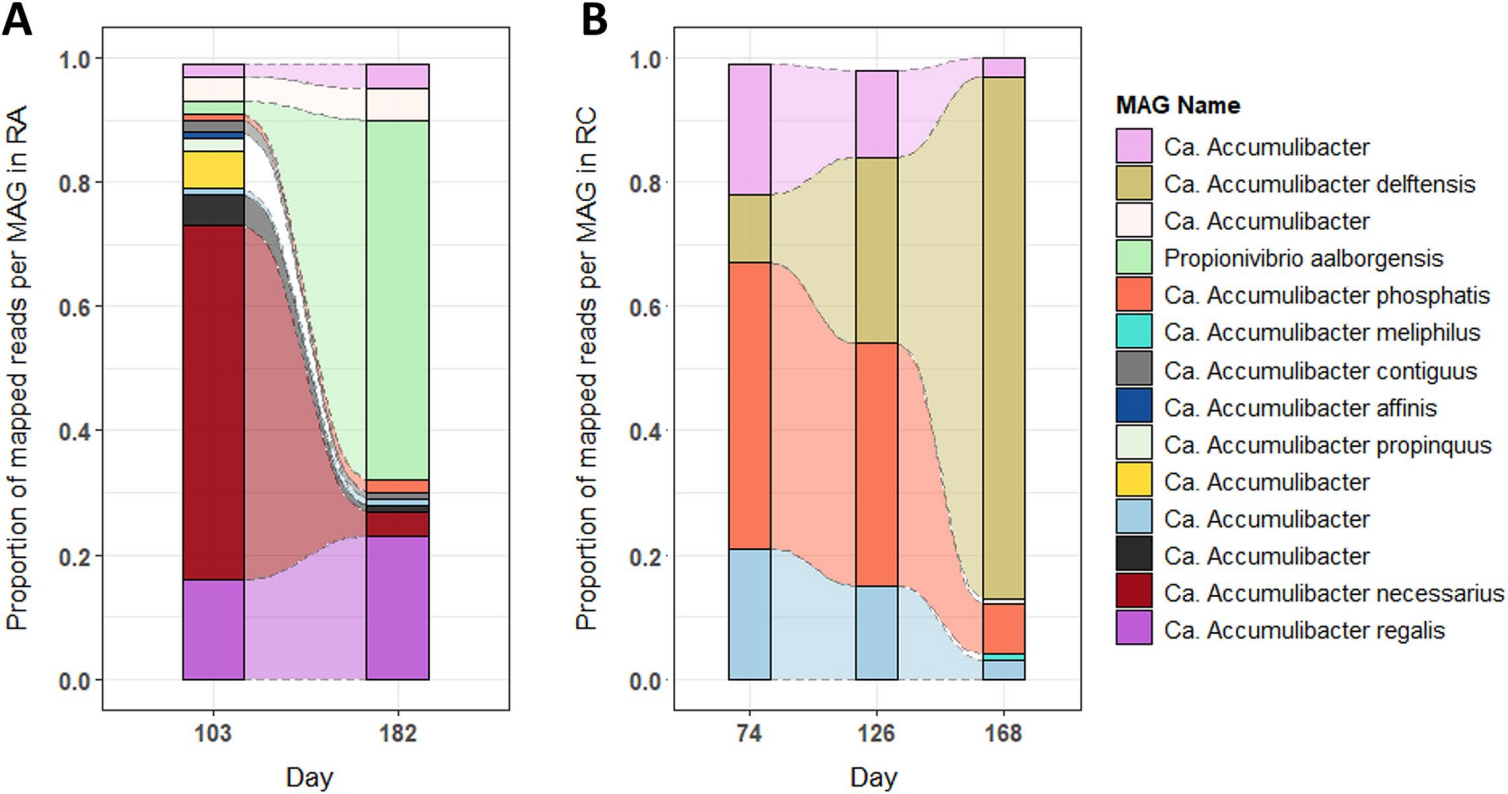
Proportion of the different MAGs at each time point in the experiments RA and RC. The figure represents the proportion of the reads mapping uniquely to the different MAGs. The raw counts were summed for each MAG in each sample, then the mean value was calculated per day (grouping the cycle and phases). **(A)** In experiment RA and **(B)** in experiment RC.

To identify the predominant *Accumulibacter* and *Propionivibrio* populations and the extent to which their respective transcriptomes were sequenced, the proportion of uniquely mapped reads and the sequencing depth of the metatranscriptomics analysis for each MAG were evaluated. Between the two experiments, a difference in *Accumulibacter* populations is observable. Before changing the phosphate concentration (Figure 2A, day 103), *A. necessarius* and *A. regalis* were the most abundant in RA, with 57 and 16% of the mapped reads, respectively, while *A. phosphatis* (46%) was the most abundant one in RC (Figure 2B, days 74 and 126). Furthermore, in RA, the reads mapped onto more MAGs compared to RC, where mainly four MAGs captured the reads. After decreasing the influent phosphate concentration, the proportion changed in both experiments. In RA, *A. regalis* remained abundant (23%), *A. necessarius* decreased to 4%, while *Propionivibrio* increased to 58% (Figure 2A, day 182). In RC, *A. delftensis* became the most abundant species with 84% of the mapped reads (Figure 2B, day 168).

To be able to compare the metabolism of *Accumulibacter* in the different conditions, a selection of the most transcriptionally active MAGs in all conditions was done by comparing the level of transcription to the number of genes transcribed by the MAG in the different samples (Supplementary Figure 4). In experiment RA, one MAG of *Accumulibacter* (*A. regalis*) and one MAG of *Propionivibrio* (*P. aalborgensis*) were selected. In experiment RC, two MAGs of *Accumulibacter* (*A. delftensis* and *A. phosphatis*) were kept for further analysis.

The different species of *Accumulibacter* and *Propionivibrio* were classified based on the Average Nucleotide Identity (ANI) analysis (Supplementary Figure 5). *A. regalis* and *A*. *delftensis*, highlighted in samples under low-phosphate conditions, are both from type I and belong to clades IA and IC, respectively. On the other hand, *A. phosphatis* was classified as type IIA.

### 3.3 Transcriptomic profiles of *Accumulibacter* species under high- and low-phosphate conditions

Table 1 represents the number of genes present and transcribed for each *Accumulibacter* MAGs. *A. delftensis* and *A. phosphatis* have both more than 4,100 genes, more than *A. regalis* (3746). A total of 88% (*A. phosphatis*) to 99% (*A. delftensis* and *A. regalis*) of these genes were transcribed in any of the experiments, and 80% of the genes annotated with a KO were common between the different MAGs (Table 1 and Supplementary Figure 6). When comparing the transcription profiles of *Accumulibacter* under low- versus high-phosphate conditions in each experiment, it was observed that few genes were differentially transcribed.

**Table 1:** Number of KOs annotated and transcribed from the different species of *Accumulibacter* in RA and RC.

	*Accumulibacter regalis* (RA)	*Accumulibacter delftensis* (RC)	*Accumulibacter phosphatis* (RC)
Genes (KO)	3,764 (1856)	4,180 (1931)	4,676 (1992)
KO in common	1,573		
Transcripts (KO)	3,723 (1852)	4,156 (1927)	4,139 (1940)
KO in common	1,560		

	day 103	day 182	day 126	day 168	day 126	day 168
KO up-regulated (feeding – aerobic)	19–22	37–26	64–314	91–83	85–37	65–159

For each MAG, transcription level under low-phosphate condition was compared to the high-phosphate condition at each phase. After low-count filtering and normalization using universal marker genes, genes were considered as up-regulated if determined as is by at least two of the three methods used (limma, EdgeR, DESeq2).

**Figure 3:**
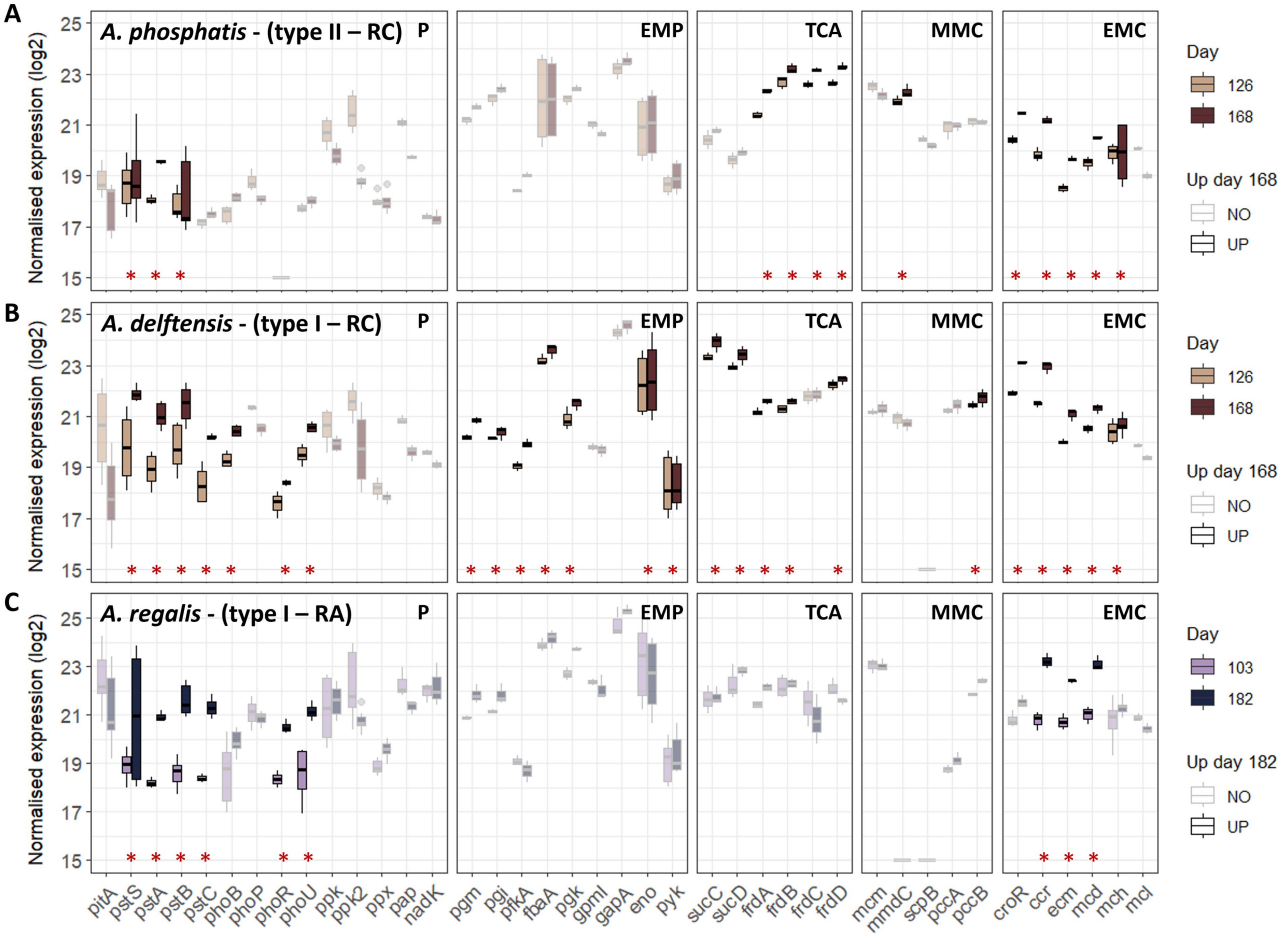
Gene transcription of the different *Accumulibacter* populations under high versus low influent phosphate concentration for both experiments RC and RA during the feeding phase. Boxplots are obtained from the log2 normalized transcription of the different transcripts associated to a specific gene in the different cycles representing biological replicates. A minimum of three values was then used to build the boxplots. However, as multiple transcripts can be found for one gene, more values can be represented for each gene (*n* ≥ 3). Genes identified as up-regulated in low- versus high-phosphate conditions are highlighted with an asterisk and bright color. **(A)** *Accumulibacter phosphatis* from experiment RC, **(B)** *Accumulibacter delftensis* from experiment RC, and **(C)** *Accumulibacter regalis* from experiment RA. Pathways are abbreviated as follow P, polyphosphate; EMP, Embden-Meyerhof-Parnas; TCA, tricarboxylic acids cycle; MMC, methylmalonyl-CoA; EMC, ethylmalonyl-CoA.

Figure 3 and Supplementary Figures 7, 8 represent the gene transcription of the pathways involved in the EBPR process for both anaerobic and aerobic phases, for the different *Accumulibacter* species selected in the previous steps. Furthermore, the genes considered as differentially transcribed in high- versus low-phosphate conditions are highlighted. The results obtained for both phases provided similar results, but the level of transcription in the anaerobic and aerobic phases were different which could indicate a difference of transcription between the phases in each condition. However, the analysis was not done to compare the gene transcription between phases, as the main focus of the experiment was the comparison between high- and low-phosphate conditions. Nonetheless, based on previously reported results (Oyserman et al., 2016), a direct comparison was made of gene transcription between phases; some differences should be expected in these experiments, too.

From the pathways involved in the EBPR process, it can be observed that only a few of them were affected by the decrease in the phosphate concentration. Mainly, the phosphate metabolism, a part of the tricarboxylic acid (TCA) cycle, the methylmalonyl (MMC) and ethylmalonyl-CoA (EMC) metabolisms presented a higher number of up-regulated genes in low- versus high-phosphate conditions, while the glycogen branching-debranching, the TCA cycle, the PHA biosynthesis-degradation, the VFA activation, or the glyoxylate shunt were not significantly affected. More specifically, *Accumulibacter* relied on the high-affinity phosphate transporter under low-phosphate conditions and on the pit system under high-phosphate conditions, as shown by the higher transcription of the *pstSABC* genes and the pho regulator system. More phosphate-related genes were affected in type I populations compared to type II, which additionally showed lower transcription of *pstSABC*.

From the pathways potentially involved in balancing the redox under low-phosphate condition, *Accumulibacter* seemed to rely more on the ethylmalonyl-CoA (EMC) pathway as most of the genes were up-regulated. Conversely, *Accumulibacter* lacks some genes of the methylmalonyl-CoA (MMC) pathway and did not show significant differences in gene transcription. An exception can be made for *Accumulibacter phosphatis* type II, which showed a higher transcription of the methylmalonyl-CoA decarboxylase (*mmd*), responsible for the production of the propionyl-CoA under low-phosphate conditions.

Only *A. delftensis* (type I) from experiment RC presented differences in the transcription of the genes related to the Embden-Meyerhof-Parnas pathway including phosphoglycerate mutase (*pgm*), phosphoglycerate kinase (*pgk*), enolase (*eno*), pyruvate kinase (*pyk*) and the VFA transporter (*actP*).

### 3.4 Transcriptional activity of *Accumulibacter* type I versus type II

**Figure 4:**
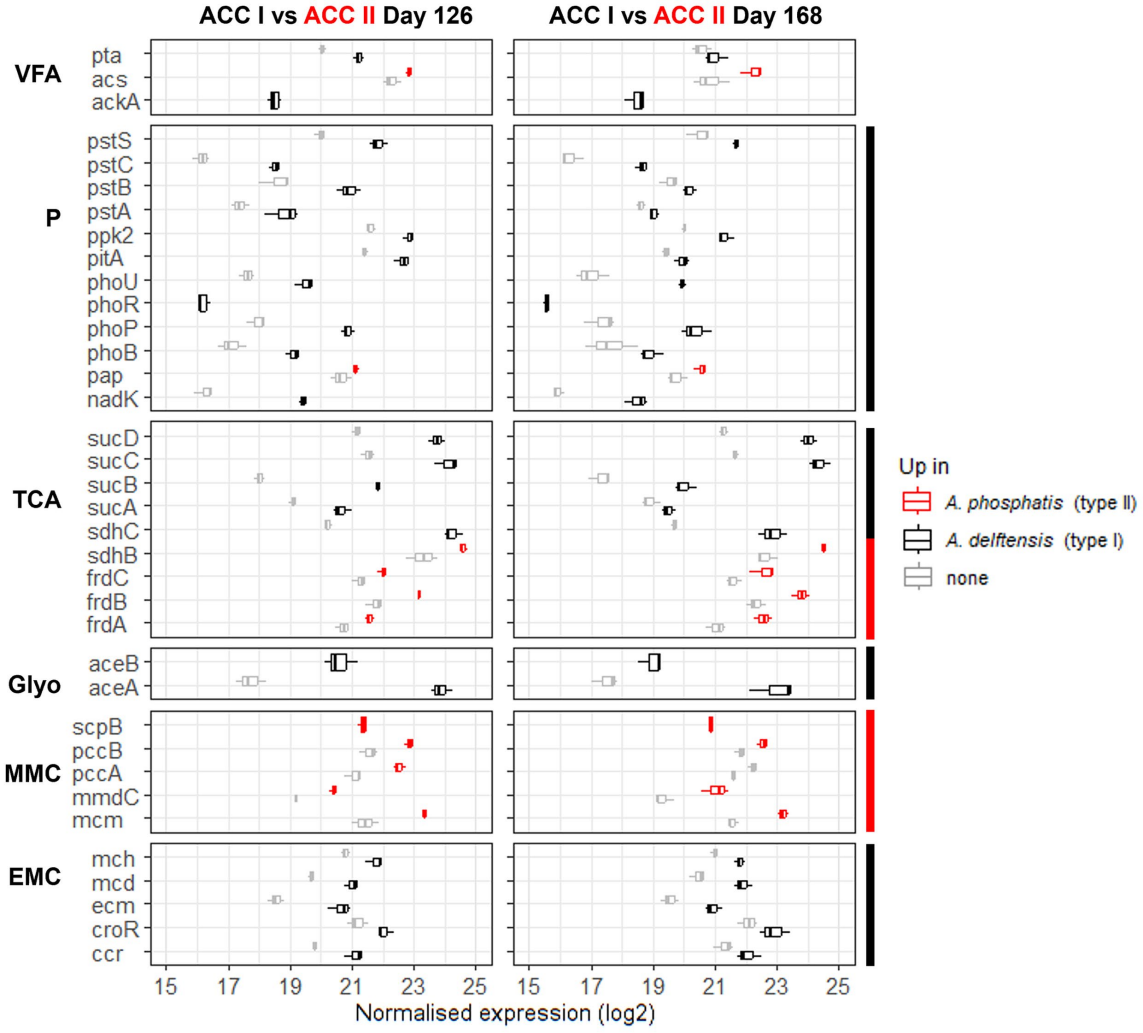
Transcription level of genes significantly up-transcribed in either *Accumulibacter delftensis* (type I) or *Accumulibacter phosphatis* (type II) under high- (day 126) or low-phosphate (day 168) conditions in experiment RC in the feeding phase. The transcription of each KO were summed up to compare both microbes and the boxplots are obtained from the values in the different cycles (*n* = 3). The genes transcription level for *Accumulibacter phosphatis* are colored in red and in black for *Accumulibacter delftensis*. The genes up-transcribed for *Accumulibacter phosphatis* are highlighted in red and the genes up-transcribed for *Accumulibacter delftensis* in black. Pathways or part group of genes constantly different between the two MAGs are highlighted with a side color bar (red for *A. phosphatis* and black for *A. delftensis*). Pathways are abbreviated as follows: VFA, volatile fatty acids; P, polyphosphate; TCA, tricarboxylic acid cycle; Glyo, glyoxylate shunt; MMC, methylmalonyl-CoA; EMC, ethylmalonyl-CoA.

In experiment RC uniquely, *Accumulibacter* type I was compared to *Accumulibacter* type II under high- (day 126) or low-phosphate (day 168) conditions. In order to compare the different transcription profiles of the two MAGs, the analysis was conducted at the gene ortholog levels. Table 2 summarizes the number of differentially transcribed KO in the different comparisons. An orthologs was defined as up or low-transcribed if it was differentially transcribed in either *A. delftensis* or *A. phosphatis.* The results are summarized in Figure 4 for the feeding phase and in the Supplementary Figure 9 for the aerobic phase.

**Table 2:** Number of KOs up-transcribed in either *Accumulibacter delftensis* type I or *Accumulibacter phosphatis* type II under different phosphate conditions for each phase.

	*Accumulibacter delftensis*		*Accumulibacter phosphatis*	
	day 126	day 168	day 126	day 168
KO up-transcribed (feeding-aerobic)	694–770	718–710	629–632	644–710
Common in both phases	649	643	554	578

After normalization using universal marker genes, transcripts were summed up to the associated KO number before using EdgeR for the differential gene transcription analysis.

It can be noticed that the differences in the gene’s transcription between the *Accumulibacter* types were the same in both phosphate conditions, suggesting an inherent difference between the types. Compared to *Accumulibacter phosphatis* type II, *Accumulibacter delftensis* type I up-transcribed the genes from the phosphate-related pathway, the EMC, the VFA activation *via* the phosphate acetyltransferase (*pta*) and the acetate kinase (*ackA*), the *sucABCD* genes from the TCA cycle, and the glyoxylate shunt. Conversely, *A. phosphatis* type II up-transcribed genes from the MMC pathway, the fumarate reductase (*frdABCD*) and succinate dehydrogenase iron–sulfur subunit (*sdhB*) from the TCA cycle, and the VFA activation *via* the acetyl-coenzyme A synthetase (*acs*) but only in the high-phosphate condition.

### 3.5 Transcriptional activity of *Propionivibrio* versus *Accumulibacter*

To elucidate how the transcriptomes of GAO *Propionivibrio* and *Accumulibacter* – which can display GAM and PAM – differed, and whether *Accumulibacter* performing GAM relied on different pathways than the GAO *Propionivibrio,* differential expression analysis was conducted at the gene ortholog levels. Table 3 and Supplementary Figure 10 indicate that around 80% of the KO transcribed by *Propionivibrio* were also transcribed by *Accumulibacter*.

**Table 3:** Number of KOs annotated and transcribed from *Accumulibacter* and *Propionivibrio*.

	*Accumulibacter regalis*	*Propionivibrio aalborgensis*
Genes (KO)	3,764 (1856)	3,667 (1818)
KO in common	1,521	
Transcripts (KO)	3,723 (1852)	3,620 (1817)
KO in common	1,518	

	day 103	day 182	vs day 103	vs day 182
KO up-transcribed (feeding-aerobic)	584–571	497–523	788–768	536–495
Common in both phases	501	441	682	455

After low-count filtering and normalization using universal marker genes, transcripts were summed up to the associated KO number before using EdgeR for the differential gene transcription analysis.

The analysis was done to compare the transcription profile of the GAO *P. aalborgensis* to *A. regalis* when the latest one was under high-phosphate condition, considered behaving as a classical PAO (day 103), or under low-phosphate condition, considered to behave as a GAO (day 182). In both cases, *Propionivibrio* transcription was taken from day 182 as the sequencing depth was not high enough under high-phosphate conditions at day 103. Feeding and aerobic phases were compared separately.

The number of differentially transcribed KOs was lower when comparing *Propionivibrio aalborgensis* to *Accumulibacter regalis* under GAM mode than compared to *Accumulibacter* under PAM mode (Table 3). Moreover, only few of these KOs, 47 and 55 in feeding and aerobic phases respectively, were different when comparing *Propionivibrio* to *Accumulibacter*-GAM (Supplementary Figures 11A,B). Interestingly, the number of differentially transcribed KOs determined when comparing two types of *Accumulibacter* in GAM mode was higher than the number obtained when comparing *Accumulibacter*-GAM to the GAO *Propionivibrio* (Supplementary Figures 11C,D). However, at least half of these KOs were shared between the two comparisons.

**Figure 5:**
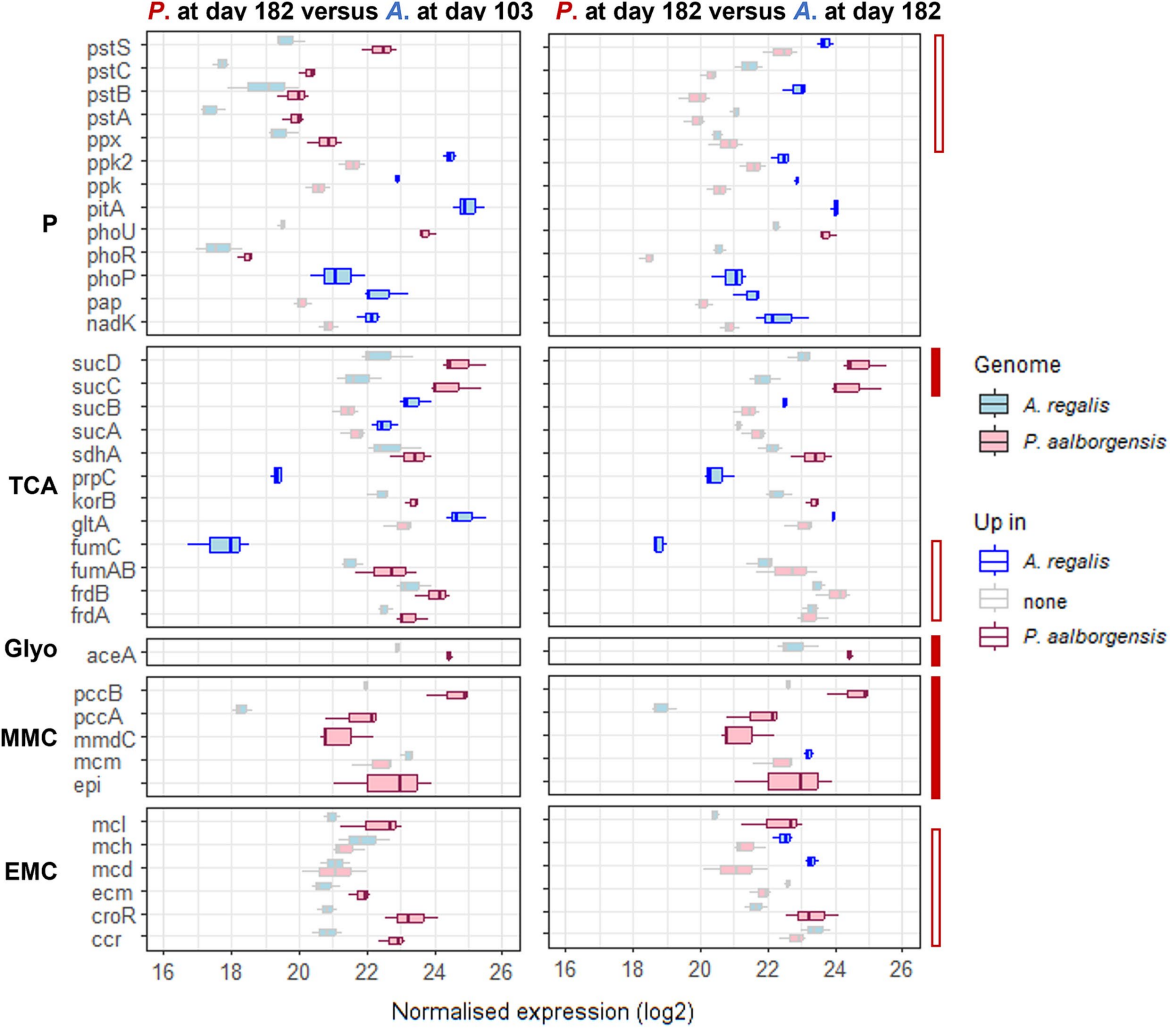
Transcription level of the genes significantly up-transcribed by either *Propionivibrio aalborgensis* or *Accumulibacter regalis* under high- (day 103) and low-phosphate (day 182) conditions in experiment RA in the feeding phase. The transcription of each KO was summed to compare both microbes, and the boxplots are obtained from the values in the different cycles (*n* = 3). The gene transcription level for *Propionivibrio aalborgensis* is colored in red, and in blue for *Accumulibacter regalis*. The up-regulated genes in one or the other species are highlighted with a bright color. The plain-red bar highlights the genes always up-regulated in *P. aalborgensis* while the empty-red bar highlights the genes with a different transcription in the two phosphate conditions. Pathways are abbreviated as follow P, polyphosphate; TCA, tricarboxylic acid cycle; Glyo, glyoxylate shunt; MMC, methylmalonyl-CoA; EMC, ethylmalonyl-CoA.

Some transcriptional differences were observed in the EBPR-related pathways between *Propionivibrio* and *Accumulibacter*, as shown in Figure 5 and Supplementary Figure 12. *Propionivibrio* exhibited a higher transcription of the reductive TCA cycle (*frd* and *suc* genes) associated with the complete transcription of the MMC pathway compared to *Accumulibacter* in either high- or low-phosphate conditions. Conversely, *Accumulibacter* up-transcribed genes from the phosphate pathway, which would be expected as *Propionivibrio*, although needs to incorporate phosphate for survival, do not rely on the polyphosphate reserve for energy production. An exception was observed for the *pst* transporter and pho regulon which were up-transcribed by *Propionivibrio* when compared to *Accumulibacter* under high-phosphate condition. However, this could be explained by the low transcription of the high-affinity transporter in *Accumulibacter* when the concentration of phosphate is high, as described in section 3.3.

## 4 Discussion

### 4.1 Effect of low-phosphate conditions on *Accumulibacter* population dynamics

This study focused specifically on *Accumulibacter* species dynamics and metabolisms under different phosphate conditions. In order to optimize the enrichment of different *Accumulibacter* species, two SBRs with different media compositions were operated. Acetate and propionate or acetate and glucose were used as carbon sources in experiments RA and RC, respectively. Both volatile fatty acids, especially propionate, associated with a low COD/PO_4_-P ratio, were previously acknowledged to favor the enrichment of *Accumulibacter* (Weissbrodt et al., 2013). The glucose uptake by *Accumulibacter* remains unclear, although some studies have suggested that some species are capable of utilizing glucose (Ziliani et al., 2023; Xie et al., 2024a). In addition to the carbon sources as a selective agent, allylthiourea (ATU) was added in experiment RA to inhibit the nitrification and subsequently the denitrification. It is well recognized that the denitrification capability differs between *Accumulibacter* species (Skennerton et al., 2015; Saad et al., 2016). The absence of ATU could have allowed the selection of denitrifying *Accumulibacter*.

The difference in the operational conditions could explain the observation of different *Accumulibacter* species between the two reactors. The lowest diversity in species observed in RC compared to RA can be due to the longer duration of the experiment and/or the medium composition. However, it is impossible to disentangle the influence of these different elements in the selection process due to the absence of control.

Nonetheless, the results indicate the persistence of *Accumulibacter* type I under low-phosphate conditions. If previous reports suggest that both *Accumulibacter* type I and II can shift their metabolisms from PAM to GAM, in most of these studies, mainly *Accumulibacter* type II remained the most abundant under low-phosphate concentration (Acevedo et al., 2012; Welles et al., 2015, 2016). One possible explanation given by Welles et al. (2015) was that *Accumulibacter* type I demonstrates a lower competitiveness than type II due to a reduced acetate uptake rate under polyphosphate-depleted conditions (Welles et al., 2015). However, when a longer acclimation period to the phosphate modification was applied, as done in the present study and by Acevedo and coworkers, *Accumulibacter* type I became the most active. The authors suggested that the acclimation period and the pH, which was not regulated and varied from 7 to 9, could influence the distribution of the different *Accumulibacter* types to shift from PAM to GAM (Acevedo et al., 2017). The pH is a factor well recognized to influence the kinetics of the carbon uptake between the microorganisms (Zhang et al., 2007; Weissbrodt et al., 2013). Furthermore, the potential use of different pathways between the two types can explain the different adaptation capabilities of *Accumulibacter* types. *Accumulibacter* type I enhancing the low-affinity acetate activation, phosphate-related pathway and the EMC pathway for redox balancing, while *Accumulibacter* type II seemed to rely more on the MMC pathway. These results are described in more detail in the following sections.

In experiment RA, the GAO *Propionivibrio* became highly abundant under low-phosphate conditions. In previous studies, no GAO were detected in high abundance in high- or low-phosphate conditions. However, FISH probes targeting some other GAOs, such as *Defluviicoccus* and *Candidatus* Competibacter, were used to estimate their abundance, albeit specific probes targeting *Propionivibrio* were not used. Moreover, the use of the PAO-mix probes (PAO492, PAO651, PAO846) can be misleading, as these probes target *Propionivibrio,* as shown by (Albertsen et al., 2016). The enrichment of *Propionivibrio* in the current study could have been the effect of the soft conditioning cycle operated on the RA reactor, i.e., settling and withdrawal operated before the aerobic phase for two cycles repeated twice in two weeks, whereas no conditioning cycle was operated in RC. In their studies, Zhao et al. (2022, 2023) operated their reactors with these inverted phases over an extended period. In their case, they obtained an enrichment of the GAO *Candidatus* Contendobacter, which may have been favored over the other GAO by the longer period of operation and/or different operational conditions (e.g., acetate as sole carbon source, non-controlled pH).

Our results showed that not only the pH or the duration of the experiment, but also the carbon source, the presence or absence of ATU, and the flanking community members (i.e., presence of GAO), can play a role in *Accumulibacter* type distribution under low-phosphate conditions. These different operational parameters can possibly trigger specific metabolic activities and should not be neglected while interpreting the metatranscriptomics results.

### 4.2 PAMs and GAMs pathways of *Accumulibacter* and *Propionivibrio*

**Figure 6:**
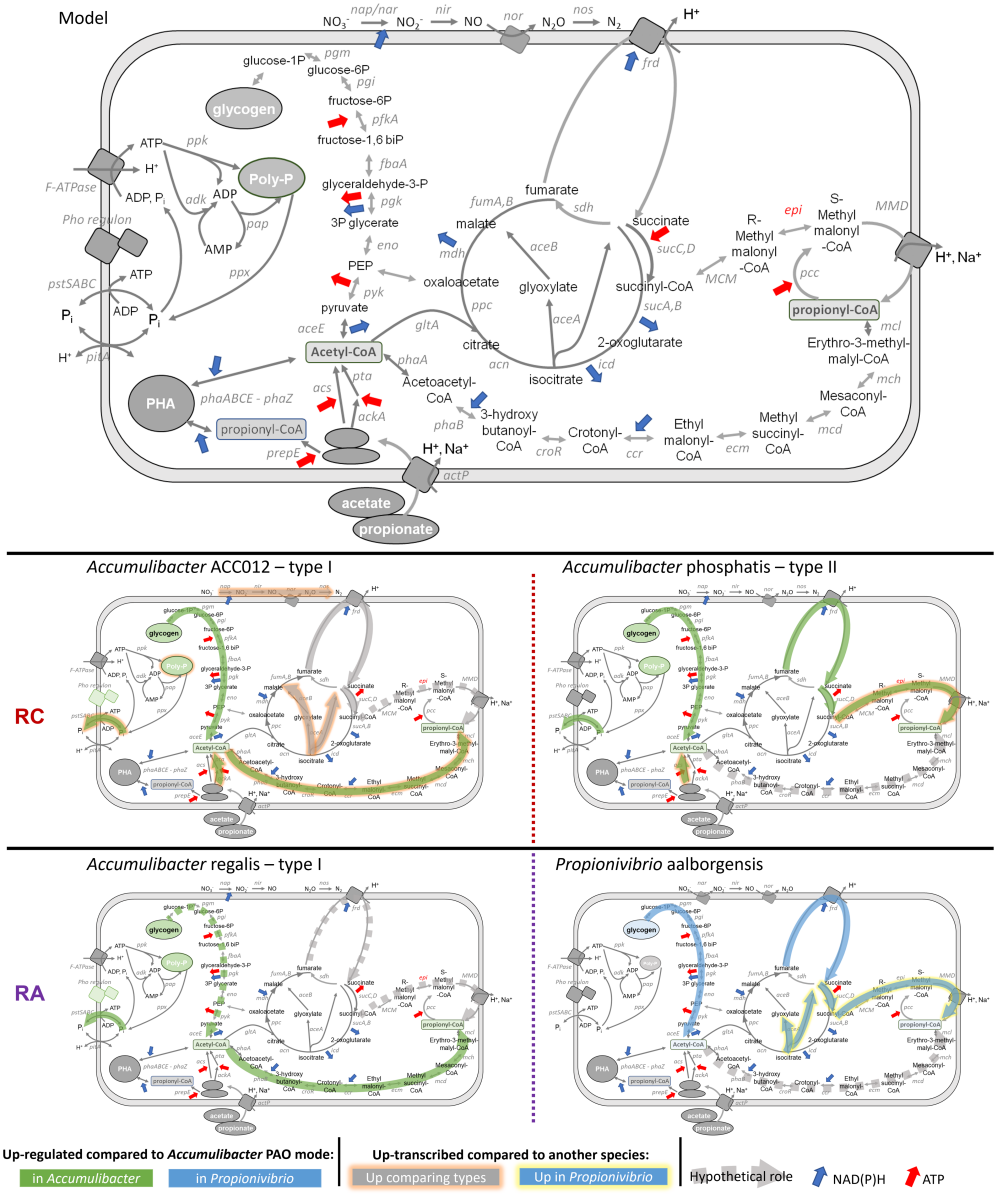
Schematic of the metabolism of *Accumulibacter* and *Propionivibrio* during the feeding phase and low-phosphate condition. Highlighted in green, or blue for *Propionivibrio*, are the pathways up-regulated in *Accumulibacter* species under low-phosphate compared to high-phosphate condition. Highlighted in orange in *Accumulibacter* or yellow in *Propionivibrio* are the pathways up-transcribed in one species compared with another. Dashed line highlights potential pathways used under low-phosphate condition (transcribed but not significantly differentially transcribed between phosphate conditions): Arrows indicate release/consumption of energy (red) and reducing equivalents (blue).

The metabolisms of the different populations were compared under the different conditions and discussed below. A schematic of the transcription profiles of *Accumulibacter* under different phosphate conditions and *Propionivibrio* is proposed in Figure 6 and Supplementary Figure 13.

#### 4.2.1 Carbon uptake and activation

In both experiments, the carbon sources were removed during the anaerobic phase, meaning that acetate, propionate, and glucose were consumed. *Accumulibacter* and *Propionivibrio* are known to be able to take up acetate and propionate *via* the acetate symporter (*actP*) and further activate to acetyl-CoA *via* two different ways: the high-affinity acetyl-CoA synthase (*acs*) or the low-affinity acetate kinase A (*ackA*) combined with phosphotransacetylase (*pta*). Although both pathways were shown to be expressed, metaproteomics analysis on *A. phosphatis* showed a higher expression of the ACS protein, suggesting a preferential use of the high-affinity activation pathways (Wilmes et al., 2008). At the transcriptional level, it seemed that the preferential pathway for VFA activation was different between *Accumulibacter delftensis* type I, which up-transcribed *ackA* and *pta*, and *Accumulibacter phosphatis* type II, which up-transcribed *acs*. The use of the low-affinity pathway by *Accumulibacter* type I could explain the lower competitiveness of *Accumulibacter* type I under drastic phosphate limitation, as described by Welles et al. (2015).

The capacity of *Accumulibacter* to directly take up and store glucose is still unclear. Recently, Ziliani et al. (2023) reported an *Accumulibacter*-enriched biomass reactor efficiently operating biological phosphorus removal with glucose as the sole carbon source. Based on stochiometric, metagenomics, and metaproteomics results, the authors hypothesized that *Accumulibacter* could directly store glucose as glycogen and partially as PHA. However, they did not exclude the potential role of an ancillary microorganism to ferment glucose into acetate. A more recent study done by Xie et al. (2024a) recovered new clades of *Accumulibacter* using metagenomics analysis. The authors identified two genomes carrying out the glucose uptake gene *ptsG* (K02779) probably acquired *via* horizontal transfer and hypothesized that the ability of *Accumulibacter* to take up and utilize glucose is species specific. In the present study, three MAGs of *Accumulibacter* carried the *ptsG* gene, but none of them were part of the studied species. Although the studied species transcribed all necessary genes for glucose utilization, especially glucokinase *glk* (K00845), EMP and PHA pathways, as suggested in Ziliani et al. (2023), it is not enough to clearly state the direct utilization of glucose by *Accumulibacter*. Further analyses, such as batch tests, are needed to decipher the role of *Accumulibacter* and the presence of fermenters that could interact with *Accumulibacter* in glucose utilization.

#### 4.2.2 Phosphate uptake and regulation

Phosphorus is an essential element for biological processes in bacteria. Genes involved in the P cycling are composed of transporters (*pit* and *pst* transport systems), poly-P synthesis (polyphosphate kinase *ppk*), hydrolysis (exopolyphosphatase *ppx*), and regulators (*pho* regulator operon). Some of these genes are very common in bacteria, even in non-PAO. The low-affinity phosphate transporter *pitA* exhibits limited distribution among bacteria, although it can be observed in certain GAO, such as *Defluviicoccus* (Maszenan et al., 2022) and some genomes of *Propionivibrio* (Petriglieri et al., 2022).

##### 4.2.2.1 High-affinity transport system *pst*

The phosphate regulon is involved in the regulation of inorganic phosphate transport into the bacterial cell. When the phosphate concentration is low, inner-membrane histidine kinase *phoR* is activated and induces the phosphorylation of the cytoplasmic transcriptional response regulator *phoB* (also called *phoP*), which in turn activates the transcription of the *pho* regulon and the *pst* transport system (Santos-Beneit, 2015; Baek and Lee, 2024). In the present study, both *pho* regulon and *pst* transporter were up-regulated under low-phosphate condition, especially in both *Accumulibacter* type I species.

It is worth noticing that in experiment RC, the two predominant *Accumulibacter* populations displayed different levels of *pst* transcription. In reactors enriched with different clades of *Accumulibacter* where phosphate was low but poly-P was not limiting the VFA uptake, Welles and coworkers determined that *Accumulibacter* type II exhibited a partial GAO metabolism, while type I behaved as a typical PAO (Welles et al., 2015). This dual metabolism in *Accumulibacter* type II could create a lower dependency on phosphate and could explain the lower transcription of *pst* compared to type I.

Compared to the proper GAO *Propionivibrio* (Albertsen et al., 2016), *Accumulibacter regalis* exhibited a higher level of transcription of the phosphate pathways. *Propionivibrio* does not use poly-P for ATP generation, but it still needs phosphate biosynthesis, which explains the observed gene transcription from polyphosphate metabolism and the differences with *Accumulibacter* type I, which seemed to depend on phosphate as mentioned previously.

##### 4.2.2.2 Low-affinity transport system *pit*

The efflux of inorganic phosphate through the pit system occurs in symport with a proton, which has been identified as the primary source for generating the proton motive force (PMF) that drives VFA uptake in *Accumulibacter* (Saunders et al., 2007). In the absence of *pitA*, as observed for *P. aalborgensis* in our study, it has been postulated that the PMF is generated *via* the export of protons through the F1F0-ATPase, the activity of the fumarate reductase (*frd*) associated with the reductive TCA cycle, and/or the methylmalonyl-CoA carboxylase forming propionyl-CoA (Albertsen et al., 2016). The transcription of *pitA* and genes related to poly-P formation was higher under high-phosphate condition while the transcription of *frdAB* was enhanced under low-phosphate condition for both *Accumulibacter* species in experiment RC. These findings suggest that *Accumulibacter* utilized polyphosphate for anaerobic VFA uptake when phosphate was abundant, but relied on the fumarate reductase to activate the PMF under low-phosphate conditions, in a manner analogous as described for the GAO *Propionivibrio*. However, in the case of experiment RA, the level of transcription of the *frd* genes were not defined as differentially transcribed although *frdA* and *B* transcription were slightly higher under low-phosphate compared with high-phosphate condition. Complementary analyses, such as carbon uptake assays, as presented by (Chen et al., 2022), using different inhibitors (F1F0-ATPase inhibitor *N, N,*-dicyclohexylcarboiimide or fumarate reductase inhibitor oxantel) combined with omics such as proteomics and metabolomics, could help drawing a better picture of the pathways used by these different MAGs under PAM and GAM mode.

#### 4.2.3 Anaerobic redox balance

Under low-phosphate conditions, the carbon sources were taken up during the anaerobic phase, while no typical phosphate release was observed. Moreover, as explained previously, fumarate reductase suggested to compensate for the activation of the PMF for VFA uptake was enhanced under low-phosphate conditions. These results strongly suggest a shift from PAM to GAM of both *Accumulibacter* types. However, stochiometric and metabolite analyses of the glycogen and PHA would have confirmed the GAM phenotype.

Utilizing glycogen as the primary source of ATP would result in an excessive production of reducing equivalents. In GAO, various pathways consuming these reducing equivalents have been proposed to offset their overproduction (McIlroy et al., 2014; Albertsen et al., 2016; Guedes da Silva et al., 2020; Maszenan et al., 2022). The most commonly described pathway involves the reductive TCA cycle in conjunction with the methylmalonyl-CoA (MMC) pathway. In this pathway propionyl-CoA is produced leading to a higher synthesis of PHV. The anaplerotic ethylmalonyl-CoA (EMC) pathway was suggested as a potential alternative pathway for redox balancing in *Defluviicoccus* (Maszenan et al., 2022) and *Candidatus* Competibacter (McIlroy et al., 2014) based on their genome analysis. The EMC pathway results in the production of propionyl-CoA and glyoxylate from acetyl-CoA.

##### 4.2.3.1 *Accumulibacter* species differences under GAM

From our results it appears that *Accumulibacter* type II relied more on the MMC pathway compared to *Accumulibacter* type I to balance the redox balance under low-phosphate condition, although transcribing the EMC pathway too (Figure 4). These results are in line with the suggestion from Acevedo et al. (2012) that *Accumulibacter* type II functioning as GAO utilized the MMC pathway to maintain redox balance potentially more efficiently than type I species (Acevedo et al., 2012).

Conversely, *Accumulibacter* type I exhibited an increased transcription of genes associated with the EMC pathway. This observation suggests that the EMC pathway could serve as a mechanism for propionyl-CoA production and redox balance especially in *Accumulibacter* type I in both experiments RA and RC.

It is worth noticing that the EMC pathway was found to be an alternative way for glyoxylate production in organisms lacking isocitrate lyase (*aceA*), such as *Methylobacterium extorquens* (Schneider et al., 2012). However, *A. delftensis* and *A. regalis*, both type I, transcribed both isocitrate lyase (*aceA*) and malate synthase (*aceB*) at similar level across different conditions with the EMC pathway being active. Conversely, *A. phosphatis* (type II) barely transcribes *aceA* and does not transcribe *aceB*. These results are in contradiction with what was observed in *M. extorquens* and suggest a possible co-existence of the two pathways, as suggested by (McIlroy et al., 2014) and (Maszenan et al., 2022) for *Competibacter* and *Defluviicoccus*, respectively. The interaction between the glyoxylate cycle and the EMC pathway, their potential alternative roles, and their influence on the ability of *Accumulibacter* to adapt its metabolism under phosphate-limited conditions remain unclear.

##### 4.2.3.2 Comparison with GAO *Propionivibrio*

In experiment RA, *Propionivibrio* transcribed both the MMC and EMC pathways to a greater extent than *A. regalis* in both high- and low-phosphate conditions. Moreover, it differed from *Accumulibacter* in that it possessed and transcribed the epimerase gene and the methylmalonyl-CoA decarboxylase gene. Zhao et al. (2022) also observed the transcription of both MMC and EMC pathways in *Candidatus* Contendobacter (Zhao et al., 2022). However, the authors suggested a use of the EMC pathway due to the absence or the low transcription of the MMC pathway genes. It appeared that GAO has different strategies to compensate for the excess production of reducing equivalents. As *Accumulibacter* type II, *Propionivibrio* carry and transcribed the isocitrate lyase only, although at a higher level. As with *Accumulibacter*, the preferred routes for propionyl-CoA production and redox balance in *Propionivibrio* are yet to be determined.

Further research is necessary to elucidate the distinct roles of MMC and EMC pathways in various *Accumulibacter* species and *Propionivibrio*. The EMC pathway is barely mentioned as a possible route to balance the excess of reducing equivalents, as it is the case for the glyoxylate shunt and the MMC pathway (Guedes da Silva et al., 2020). Complementary analyses such as metaproteomics, metabolomics, or tracking carbon with isotopes would be needed to: (1) determine if all pathways are fully expressed to the protein level, (2) determine whether *Accumulibacter* type II relies more on the MMC pathway than type I and if this confers a competitive advantage during phosphate limitation and (3) if the potential preference of *Accumulibacter* type I for the EMC pathway is linked to its higher dependence on the phosphate metabolism. One could hypothesize that the MMC pathway, using fewer intermediates and deriving from the reductive TCA cycle, allowed a more efficient redox balance by producing more reduced forms of PHA compared to the EMC pathway, which produces glyoxylate and propionyl-CoA. Furthermore, both fumarate reductase and methylmalonyl-CoA decarboxylase were predicted to contribute to the proton motive force needed for acetate uptake, which could explain the suggested higher uptake rate under phosphate limitation of *Accumulibacter* type II compared to type I.

#### 4.2.4 Nitrogen metabolism

The genome annotation of the different MAGs of *Accumulibacter* and *Propionivibrio* indicated a difference in the denitrification pathways (Supplementary Table 9). Indeed, the different *Accumulibacter* species studied in this study possess the periplasmic nitrate reductase *napAB,* while *Propionivibrio* possesses the respiratory nitrate reductase *narGHI*. In *Accumulibacter*, both *nar* and *nap* can be found, depending on the genomes (Skennerton et al., 2015; Saad et al., 2016). Camejo and co-workers have identified the *nar* gene in one *Accumulibacter* clade IC MAG (Camejo et al., 2019). According to Moreno-Vivián et al. (1999), the role of the periplasmic gene *nap* is unclear, as only the *nar* enzyme has been correlated with enough ATP generation for microbial processes and growth. However, it can have a role in dissipating the excess of reducing equivalents by using NADH to reduce nitrate (Bedzyk et al., 1999). All MAGs possess the nitrite reductase *nirS*. From the nitric oxide reductase cluster *norCB*, only *norB* is annotated, and *norC* is missing in *Propionivibrio*, *A. delftensis*, and *A*. *phosphatis*. Finally, the three *Accumulibacter* species possess the nitrous oxide reductase (*nosZ*).

**Figure 7:**
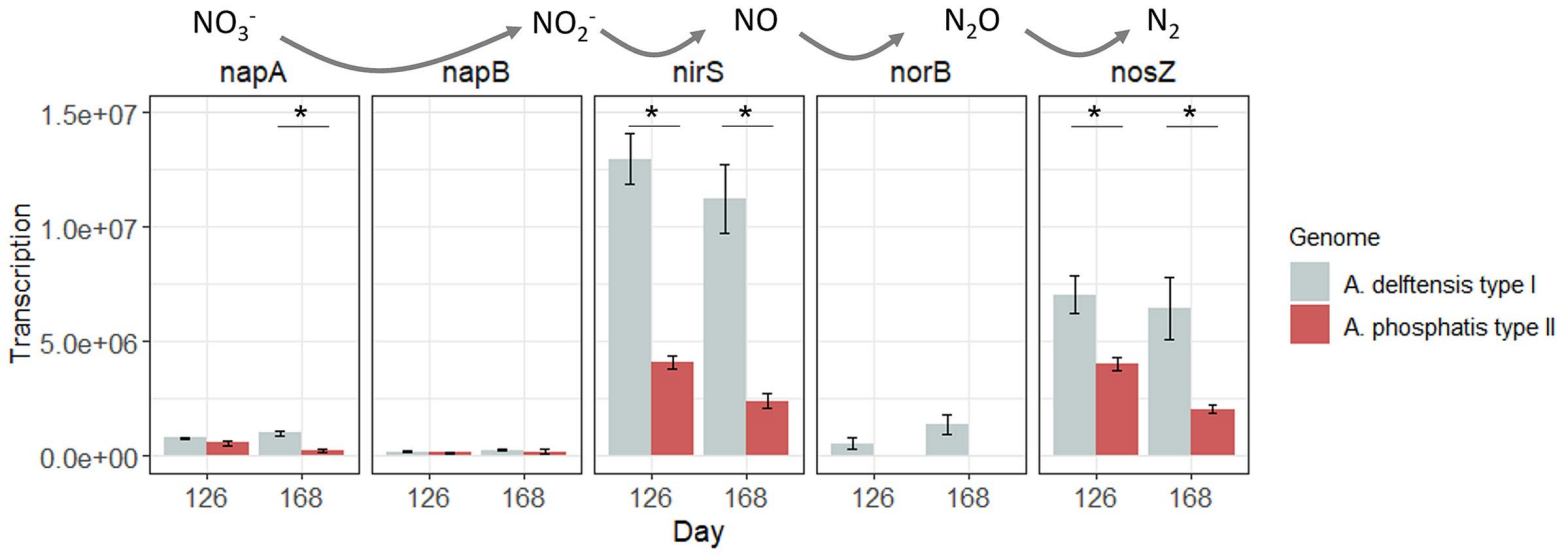
Gene transcription of the denitrification pathway for *Accumulibacter delftensis* and *A*. *phosphatis* in experiment RC. Asterix highlight significant differential transcription between *Accumulibacter* types at each time point.

Allylthiourea was added to the medium in the case of experiment RA, inhibiting nitrification and subsequently influencing denitrification. However, in the experiment, RC allylthiourea was not used. For this reason, the transcription of the genes related to denitrification was only studied in experiment RC. At the end of the experiment, under low-phosphate conditions, the nitrification seemed to be limited as the ammonium concentration slightly decreased at the end of the aerobic phase (Supplementary Figure 14). It seemed that the nitrifiers were affected by the modification of the phosphate supply. However, at the end of the anaerobic phase, there was no accumulation of nitrite or nitrate, meaning that denitrification was probably less affected. Previously, Meng et al. (2023) have evaluated the denitrifying capacity of *Accumulibacter*-enriched biomass under low-phosphate conditions (COD: P ratio of 100:1). *Accumulibacter* IA, IIC, and IID were co-enriched in a reactor and were maintained under phosphate-deprived conditions. A metabolic shift from PAM to GAM was observed based on measurements of intracellular polyphosphate, glycogen, PHA, and stochiometric results. Their results indicated that *Accumulibacter* under GAM can maintain its denitrifying capacity. However, the populations detected (*Accumulibacter*, *Dechloromonas*, and *Competibacter*) were not resolved to the species level, nor were their relative contributions to the denitrification. In our experiment, *Accumulibacter* did not show differences in the transcriptional level of the genes involved in the denitrification between high- and low-phosphate conditions (Figure 7). However, *A. delftensis* (type I) showed a higher transcription of these genes compared to *A*. *phosphatis* (type II). The influence of the denitrification capability of *Accumulibacter*, in particular the role of *nap* genes transcription in the anaerobic balance under low-phosphate conditions, needs further investigation.

## 5 Conclusion

*Accumulibacter*-enriched biomasses were obtained in two experiments operated with distinct carbon sources and with or without nitrification inhibition by allylthiourea. Despite these differences and the identification of distinct *Accumulibacter* species co-occurring in each reactor, phosphate limitation led to the enrichment of one predominant type I *Accumulibacter* species: *A. regalis* in RA and *A*. *delftensis* in RC. The comparison of the transcriptomes of different *Accumulibacter* species under high- and low-phosphate conditions highlighted the ethylmalonyl-CoA pathway as a possible alternative for the anaerobic redox balance, while the methylmalonyl-CoA pathway, always hypothesized for the production of propionyl-CoA, did not change between the different conditions. However, the results suggest a possible difference between *Accumulibacter* types, with type I enhancing transcription of the EMC, glyoxylate, denitrification, and phosphate pathways under low-phosphate conditions, while type II enhanced the MMC transcription. This study is the first of its kind, making a comparison between the metabolism of *Accumulibacter* under GAM and a classical GAO, *Propionivibrio*. The degree of difference in the transcription of EBPR-related pathways between *Accumulibacter* and *Propionivibrio* was relatively similar to that observed when comparing *Accumulibacter* from different types. *Propionivibrio* seemed to rely more on the MMC pathway, transcribing the complete set of genes at a higher level compared with *Accumulibacter regalis* (type I). As *Propionivibrio* and *Accumulibacter* are closely related phylogenetically, comparing *Accumulibacter* under GAM conditions to other GAO, such as *Competibacter*, *Defluviicoccus,* or *Contendobacter*, would provide a better understanding of the metabolisms of these microorganisms, their potential interactions, and influences on the nutrient removal performance.

## Data Availability

The datasets generated and analyzed for this study can be found in the Sequence Read Archive (SRA) repository under the BioProject ID PRJNA1238817 for the metatranscriptomic samples from experiment RC and PRJNA1144857 for experiment RA.
