# Supplementary material for: Comparative transcription profiles of Candidatus Accumulibacter and Propionivibrio under phosphate limitation in sequencing batch reactors
Source: Front Microbiol. 2025 Oct 28;16:1650167. doi: 10.3389/fmicb.2025.1650167 (PMC12604563; doi:10.3389/fmicb.2025.1650167)
Supplement: Supplementary file 13 [file Supplementary_file_1.docx]

Supplementary Material

# Supplementary Figures

**Supplementary Figure 1. Description of experiments RC and RA**


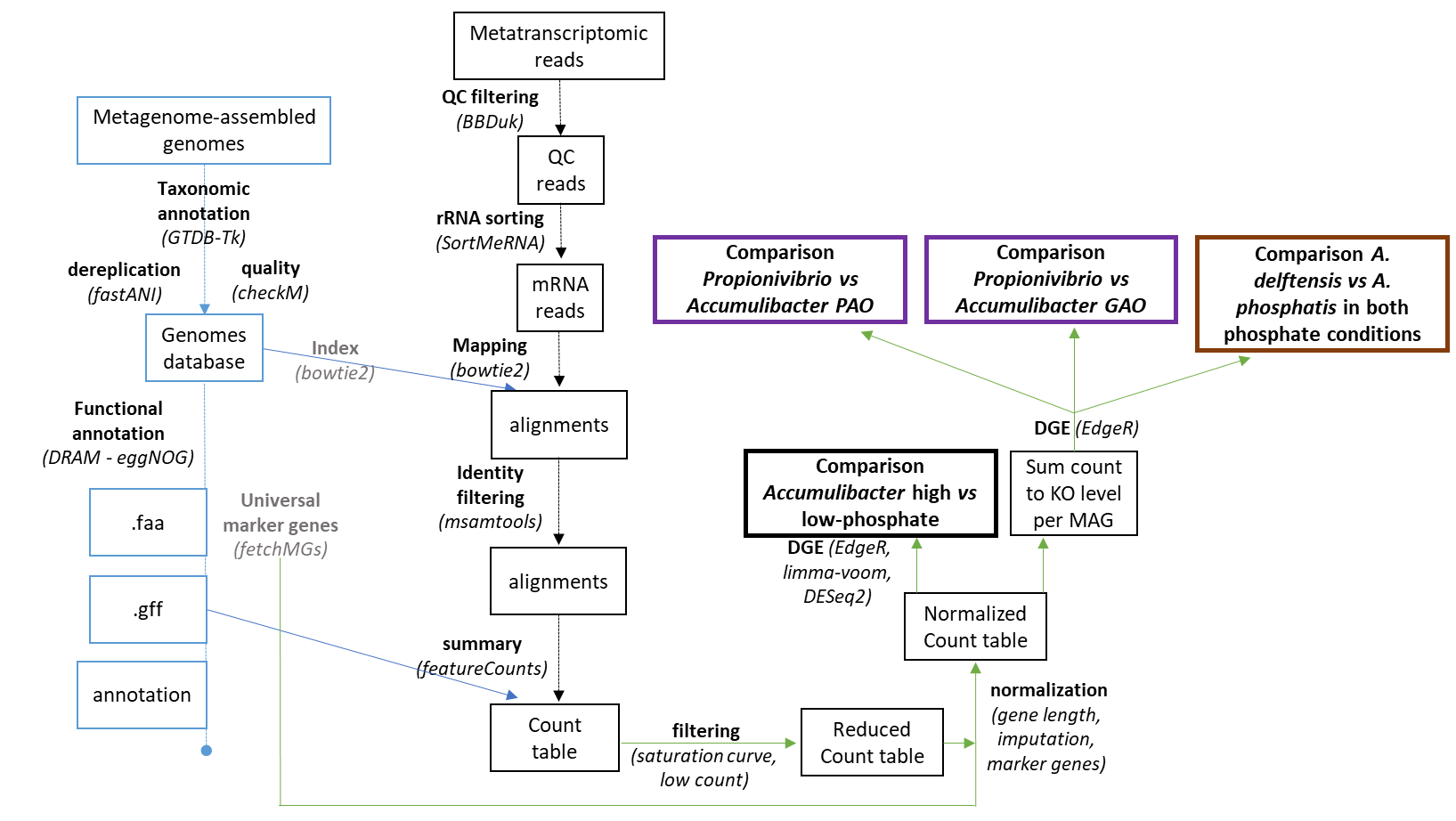


**Supplementary Figure 2. Analytical pipeline**

**Supplementary Figure 3. Number of reads kept at each step of the bioinformatic analysis. (A)** for experiment RA and **(B)** for experiment RC.


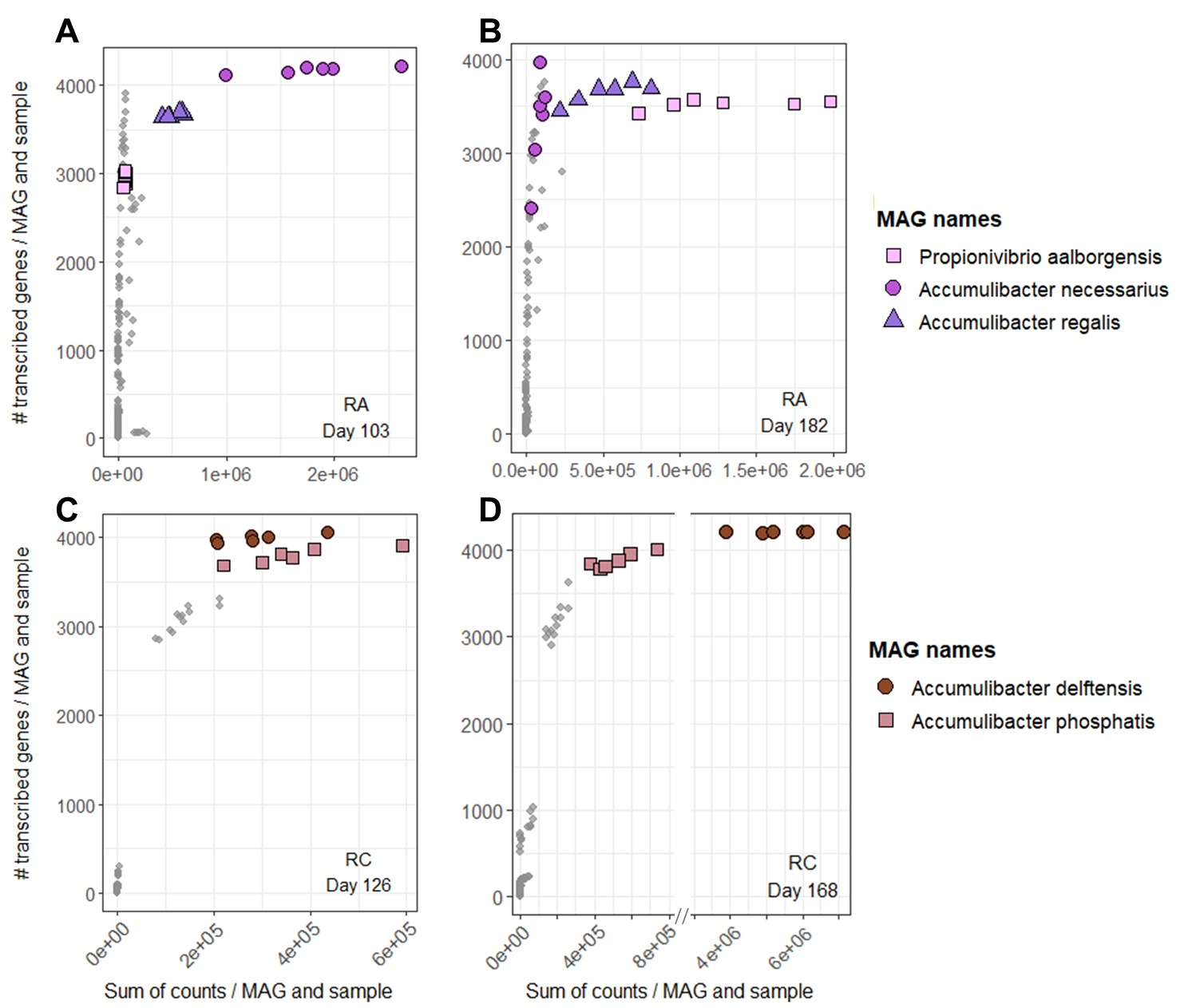


**Supplementary Figure 4. Transcription level representing the dominant genomes in experiment RA (A and B) and experiment RC (C and D).** The figure represents the number of transcribed genes per genome and sample with respect to the sum of counts per genome and sample. Only the genomes reaching the saturation in at least one sample was colored. **(A)** Results from experiment RA at day 103. **(B)** Results from experiment RA at day 182. **(C)** Results from experiment RC at day 126 and **(D)** Results from experiment RC at day 168.

Supplementary Figure 5. Average Nucleotide Identity (ANI) results between *Accumulibacter* and *Propionivibrio* metagenome assembled-genomes used to build the database of the metatranscriptomics analysis. Are highlighted with a brown square, genomes that reach saturation in RC and in purple square, those in RA.

**Supplementary Figure 6. Number of KOs in common for the different *Accumulibacter* species. (A)** from the genome annotation and **(B)** from the metatranscriptomics mapping before low-count filtering step.

**Supplementary Figure 7. Gene transcription of the different *Accumulibacter* populations under high and low influent phosphate concentration for both experiments RC and RA during the aerobic phase.** Boxplots are obtained from the log2 normalized transcription of the different transcripts associated to a specific gene in the different cycles representing biological replicates. A minimum of three values were then used to build the boxplots. However, as multiple transcripts can be found for one gene, more values can be represented for each gene (n ≥ 3). Genes identified as up-regulated in low- versus high- phosphate condition are highlighted with an asterisk and bright color. **(A)** *Accumulibacter phosphatis* from experiment RC, **(B)** *Accumulibacter delftensis* from experiment RC and **(C)** *Accumulibacter regalis* from experiment RA. Pathways are abbreviated as follow P: Polyphosphate; EMP: Embden-Meyerhof-Parnas; TCA: Tricarboxylic acids cycle; MMC: Methylmalonyl-CoA; EMC: Ethylmalonyl-CoA

**Supplementary Figure 8. Gene transcription of the different *Accumulibacter* populations under high and low influent phosphate concentration for both experiments RC and RA during the feeding or aerobic phase.** Boxplots are obtained from the log2 normalized counts of the different transcripts associated to a specific gene in the different cycles. A minimum of three values were then used to build the boxplots. However, as multiple transcripts can be found for one gene, more values can be represented for each gene (n ≥ 3). Genes identified as differentially transcribed are highlighted with an asterisk and bright color. *Accumulibacter phosphatis* from RC experiment in feeding **(A)** or aerobic **(D)** phase, *Accumulibacter delftensis* from RC experiment in feeding **(B)** or aerobic **(E)** phase and *Accumulibacter regalis* from RA experiment in feeding **(C)** or aerobic **(F)** phase. Pathways are abbreviated as follow VFA: Volatile fatty acid; Gly: Glycogen; TCA: Tricarboxylic acids cycle; PHA: Polyhydroxyalkanoate

Supplementary Figure 9. Transcription level of genes significantly up-transcribed in either *Accumulibacter delftensis* type I or *Accumulibacter phosphatis* type II under high- (day 126) or low-phosphate (day 168) conditions in experiment RC in aerobic phase. Transcription of each KO were summed up to compare both microbes and the boxplots are obtained from the values in the different cycles (n = 3). The genes up-transcribed for *Accumulibacter phosphatis* are highlighted in red and the genes up-transcribed for *Accumulibacter delftensis* in black. Pathways or part group of genes constantly different between the two MAGs are highlighted with a side color bar (red for *A. phosphatis* and black for *A. delftensis*). Pathways are abbreviated as follows: VFA: Volatile Fatty Acids; P: Polyphosphate; TCA: Tricarboxylic Acid Cycle; Glyo: Glyoxylate shunt; MMC: Methylmalonyl-CoA; EMC: Ethylmalonyl-CoA

**Supplementary Figure 10. Number of KOs in common between *Accumulibacter regalis* and *Propionivibrio aalborgensis* from experiment RA. (A)** from the genome annotation and **(B)** from the metatranscriptomics mapping before the low-count filtering step.

**Supplementary Figure 11. Number of differentially transcribed KOs in common between *Accumulibacter* populations and *Propionivibrio aalborgensis*. (A and B)** Results of edgeR analysis comparing *Accumulibacter* *regalis* and *Propionivibrio* in experiment RA, day 103 when *Accumulibacter* *regalis* behaved as a PAO and day 182 when *Accumulibacter* behaved as a GAO **(A)** in the feeding phase and **(B)** in the aerobic phase. (C and D) Results of edgeR analysis comparing *Accumulibacter phosphatis* (type II) to *Accumulibacter delftensis* (type I) at day 168 (DE-GAM type) compared to the results of edgeR analysis comparing *Accumulibacter* *regalis* (type I) to *Propionivibrio aalborgensis* at day 182 (DE-GAM species)

Supplementary Figure 12. Transcription level of the genes significantly up-transcribed by either *Propionivibrio aalborgensis* or *Accumulibacter regalis* under high- (day 103) and low-phosphate (day 182) conditions in experiment RA in aerobic phase. Transcription of each KO were summed to compare both microbes and the boxplots are obtained from the values in the different cycles (n = 3). The genes up-transcribed for *Propionivibrio aalborgensis* are highlighted in red and the genes up-transcribed for *Accumulibacter regalis* in blue. The plain-red bar highlights the genes always up-regulated in *P. aalborgensis* while the empty-red bar highlights the genes with a different transcription in the two phosphate conditions. Pathways are abbreviated as follow P: Polyphosphate; TCA: Tricarboxylic Acid Cycle; Glyo: Glyoxylate shunt; MMC: Methylmalonyl-CoA; EMC: Ethylmalonyl-CoA


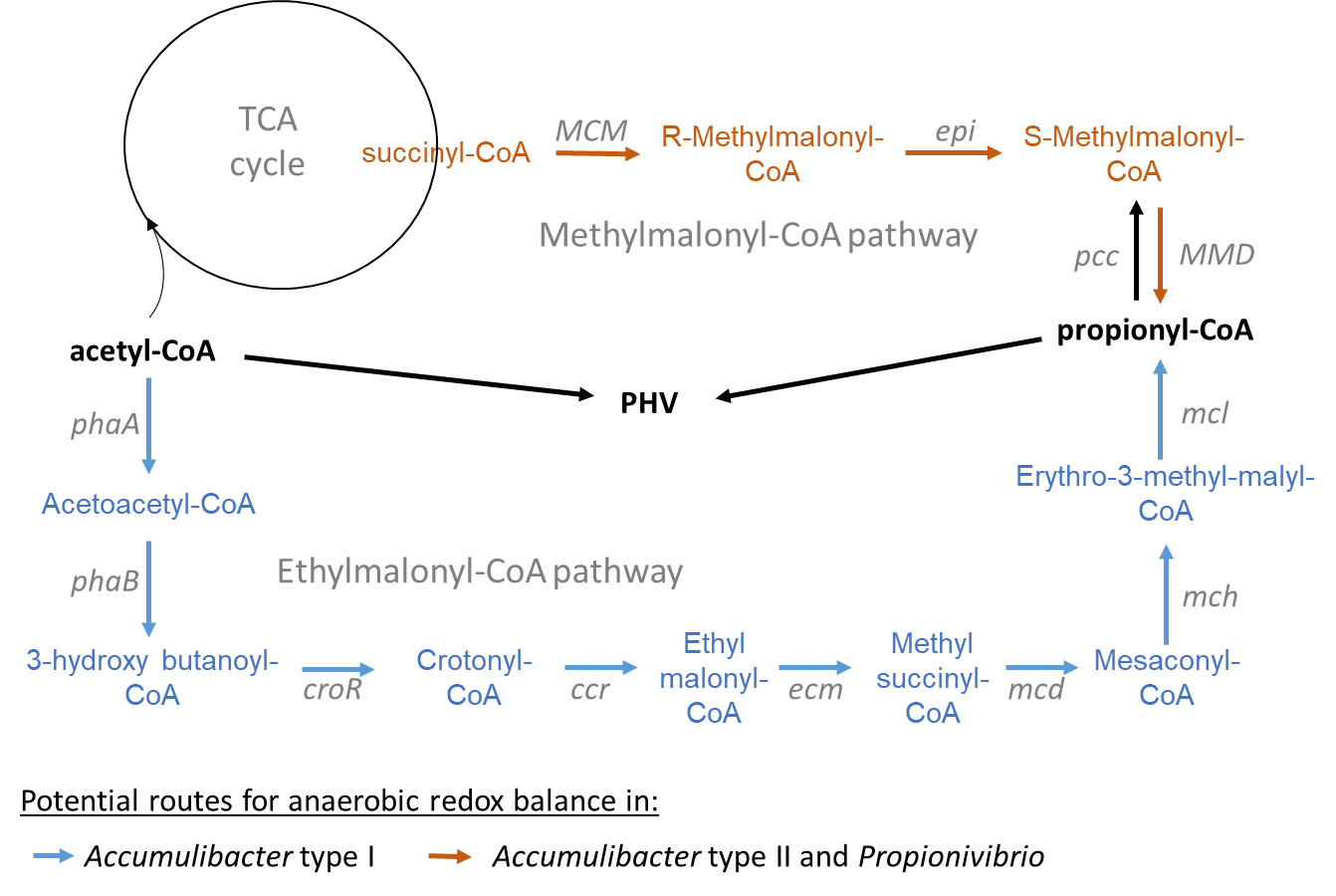


**Supplementary Figure 13. Schematic representation of both methyl- and ethyl-malonyl pathways.** The potential route used for anaerobic redox balance by *Accumulibacter* type I is highlighted in blue, and for *Accumulibacter* type II and *Propionivibrio* in orange.

**Supplementary Figure 14. Ammonium, nitrate and nitrite concentration in the influent, at the end of anaerobic and aerobic phases from experiment RC. (A)** Ammonium concentration (mg/L), **(B)** nitrate concentration (mg/L) and **(C)** nitrite concentration (mg/L). Gray zones indicate the period with decreased phosphate concentration in the influent.
